# Self-sustainable protonic ceramic electrochemical cells using a triple conducting electrode for hydrogen and power production

**DOI:** 10.1038/s41467-020-15677-z

**Published:** 2020-04-20

**Authors:** Hanping Ding, Wei Wu, Chao Jiang, Yong Ding, Wenjuan Bian, Boxun Hu, Prabhakar Singh, Christopher J. Orme, Lucun Wang, Yunya Zhang, Dong Ding

**Affiliations:** 1 Energy & Environmental Science and Technology, Idaho National Laboratory, Idaho Falls, ID 83415 USA; 2 Computational Mechanics and Materials, Idaho National Laboratory, Idaho Falls, ID 83415 USA; 3 School of Materials Science and Engineering, Georgia Institute of Technology, Atlanta, GA 30332 USA; 4 Department of Chemical & Materials Engineering, New Mexico State University, Las Cruces, NM 88003 USA; 5 Department of Materials Science & Engineering, University of Connecticut, Storrs, CT 06269 USA

**Keywords:** Solid-state chemistry, Hydrogen energy, Fuel cells, Nanoparticles

## Abstract

The protonic ceramic electrochemical cell (PCEC) is an emerging and attractive technology that converts energy between power and hydrogen using solid oxide proton conductors at intermediate temperatures. To achieve efficient electrochemical hydrogen and power production with stable operation, highly robust and durable electrodes are urgently desired to facilitate water oxidation and oxygen reduction reactions, which are the critical steps for both electrolysis and fuel cell operation, especially at reduced temperatures. In this study, a triple conducting oxide of PrNi_0.5_Co_0.5_O_3-δ_ perovskite is developed as an oxygen electrode, presenting superior electrochemical performance at 400~600 °C. More importantly, the self-sustainable and reversible operation is successfully demonstrated by converting the generated hydrogen in electrolysis mode to electricity without any hydrogen addition. The excellent electrocatalytic activity is attributed to the considerable proton conduction, as confirmed by hydrogen permeation experiment, remarkable hydration behavior and computations.

## Abstract (web summary)

While producing renewable fuel is crucial for a sustainable energy economy, there is still a need for active and durable materials capable of efficient fuel generation and utilization. Here, authors demonstrate a triple-conductive oxide as an oxygen electrode for H_2_ or electricity production.

## Introduction

**Fig. 1:**
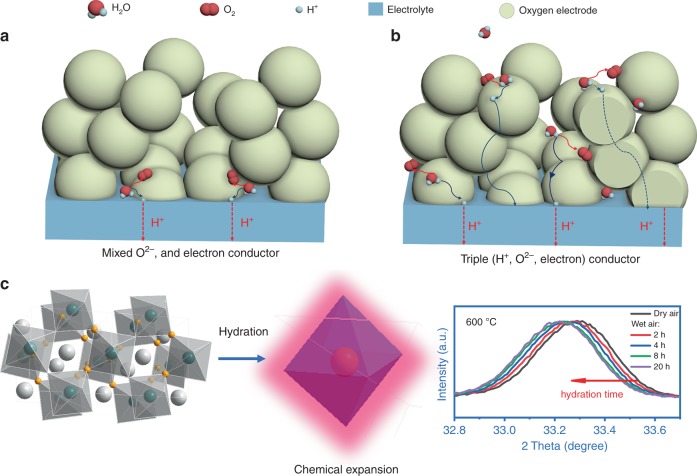
Water splitting reaction on oxygen electrode and PNC’s hydration. **a** Mixed oxygen-ion O^2−^ and electron conducting electrode. **b** Triple (H^+^, O^2−^, and electron) conducting electrode to extend reaction to entire electrode surface. **c** Chemical expansion of PNC perovskite structure observed in high-temperature X-ray diffraction at 600 °C when exposed to wet air.

The share of renewable and sustainable energy power plants has been increasing for decades in order to reduce dependency on fossil fuels and to mitigate carbon dioxide emission. Neverthess, the intermittency and variability of such an energy system creates challenges for micro-grid operators^1^. To reduce the stress on peak shaving, the development of efficient energy conversion/storage technologies is crucial. The protonic ceramic electrochemical cell (PCEC) is a proton-conductor-based solid oxide cell that can serve in a reversible operation manner to store renewable energies using water electrolysis to produce hydrogen and then convert it back to electricity in fuel cell mode^2–4^. The application of PCECs demonstrates the uniqueness of combining the bi-function of energy storage and distributed power generation by integrating PCEC and balance of the plant into one system. As significant advances have been made in solid state proton conductors and related electrochemical cells (fuel cells and electrolzyers) in the past decade^5,6^, PCEC represents a promising technology for the purpose of achieving low-cost energy storage and conversion at reduced temperatures by offering attracting advantages such as high efficiency^7,8^, longer system durability^9–11^, and less expensive materials^12,13^. However, the large-scale deployment of PCECs still remains elusive by severe limitation on developing highly active and robust electrode due to sluggish electrode kinetic at intermediate temperatures and decayed lifetime of the material and interface, especially under high-steam concentration^14,15^. The self-sustainable operation proposed in this study further demands fast electrolysis rates to produce hydrogen that can then be directly utilized for generating electricity without external hydrogen feeding. This operation imposes more rigorous requirements upon high performing and reliable material system with reversible and/or thermal cycling. One early technical opportunity is the development of oxygen electrode materials to mitigate these problems that contribute the most to performance degradation and efficiency loss in the existing PCECs^16^. Currently, both the water oxidation reaction (WOR) and the oxygen reduction reaction (ORR) in the mixed ion and electronic conducting (MIEC) electrodes are strictly confined to the triple phase boundaries (TPBs) where ion, electron, and gas meet (Fig. 1a). Therefore, in a PCEC system, it is highly desirable to introduce proton conduction into MIEC material to formulate a triple conducting oxide (TCO), i.e., electron, oxygen ion and proton, to extend TPBs from the electrolyte/electrode interface into the electrode bulk (Fig. 1b). Despite these advantages, there are very few TCO candidates developed to date due to the difficulty of creating sufficient defects for proton conduction with the prerequisite of available readily-hydrated oxygen vancancies^17,18^. Early attempts have been limited to inducing a small fraction of electronic conductivity to proton conductors by rare-earth or transition metal doping into cerates/zirconates, e.g., BaCe_0.5_Bi_0.5_O_3-δ_^19^, BaZr_0.5_Pr_0.3_Y_0.2_O_3-δ_^20^, and BaZr_0.6_Co_0.4_O_3-δ_^21^. The low total conductivity still restrains the use of these single-phase electrodes. Only recently, a few layered perovskite structured electrodes PrBa_0.5_Sr_0.5_Co_2_O_5+δ_ and NdBa_0.5_Sr_0.5_Co_1.5_Fe_0.5_O_5+δ_^18,22^ have been reported as TCOs in proton conducting fuel cells or electrolyzers at intermediate temperatures (600~700 °C), although the existence of proton conductivity needs to be further validated through dedicated experimental methods^23^.

In this article, we report our findings from the creation of a perovskite TCO electrode PrNi_0.5_Co_0.5_O_3−δ_ (PNC) in the PCECs with superior WOR and ORR activity. In addition to demonstrating excellent durability and thermal cycling capability, it shows outstanding electrochemical performances and self-sustainable and reversible PCEC at reduced temperature range (400~600 °C). PNC is inspired by PrCoO_3_ (PCO) and lanthanide nickelates. PCO is a highly active oxygen electrode in oxygen-ion conducting fuel cells without alkaline earth metal (e.g., Sr or Ba) doping^24–27^, which tends to mitigate segregation and resulting degradation during operation. Furthermore, lanthanide nickelates receives considerable interest due to their excellent tolerance against high steam vapor and broad oxygen partial pressure^28^. Our experimental study and density function theory (DFT) calculation show that proper Ni replacement in the B-sites of PCO perovskite can surprisingly reduce the migration barrier for proton conduction when proton defects are readily induced by hydration reaction, thus enhancing proton conduction. The demonstrated triple conduction of this material facilitates WOR and ORR and promoted electrochemical performance of the cell in a self-sustainable and reversible operation at reduced temperatures.

## Results

### Characterization of proton conductivity in PNC electrode

**Fig. 2:**
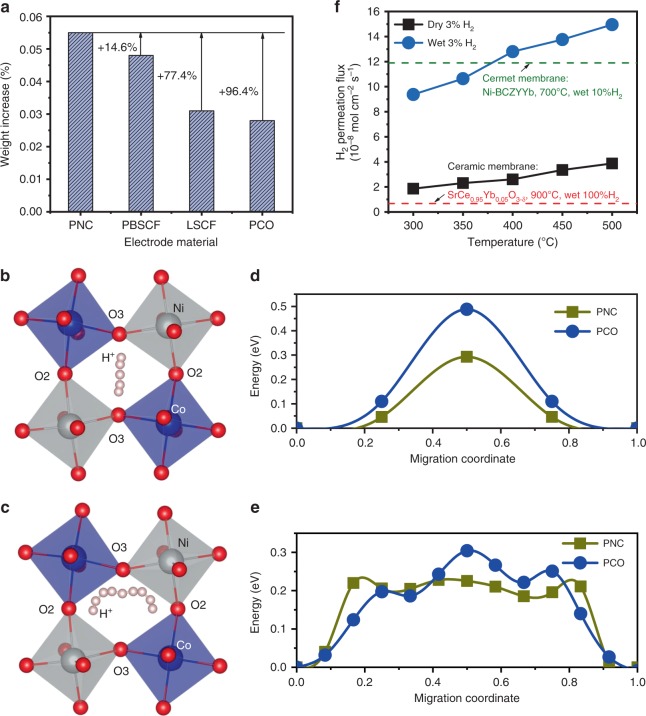
Study of hydration behavior, hydrogen permeation and proton migration energy. **a** Comparison of hydration ability for PCO, PNC, LSCF, and PBSCF electrodes at 500 °C in wet air condition. **b**, **c** Proton migration pathway I along inter-octahedron direction and pathway II along intra-octahedron direction. **d**, **e** Minimum energy paths for proton migration along pathway I and II. **f** Hydrogen permeation fluxes of PNC dense membrane.

The PNC perovskite was synthesized by the wet chemistry method and showed a single orthorhombic perovskite structure after 50% B-site replacement based on cobaltite PrCoO_3_ (Supplementary Note 1 and Supplementary Fig. 1a)^24^, space group *Pbnm*, with *a* = 5.405 Å, *b* = 5.380 Å, and *c* = 7.617 Å after Rietveld refinement (Supplementary Fig. 2)^29^. This material is stable after calcining at 600 °C in wet air (~50% H_2_O) for 200 h with absent changes on diffraction peaks. The transmission electron microscopy (TEM) and energy dispersive X-ray (EDX) analysis confirmed the presence of a highly crystalline nature and homogenous element distribution (Supplementary Fig. 1b–f). The oxygen deficiency was determined to be 0.085 at 600 °C with iodometric titration method (Supplementary Fig. 3). The chemical compatibility between the PNC and electrolyte was examined and the results didn’t show any reaction at 1000 °C (Supplementary Fig. 4). X-ray photoelectron spectroscopy, DFT calculations and thermogravimetry analysis (TGA) were used to understand the effect of Ni doping on oxygen vacancy formation and hydration behavior in the wet condition and proton migration energy. The majority valence of Ni dopant is shown to be +2, contributing to intrinsic oxygen vacancy concentration (Supplementary Fig. 5), indicating the multiple valence of A-site Pr. We have performed non-spin polarized DFT calculations to obtain the formation energies of oxygen vacancies in both PCO and PNC with an orthorhombic *Pbnm* structure (Supplementary Note 2 and Supplementary Fig. 6c). The symmetrically distinct oxygen sites in PCO (O1 and O2) and PNC (O1, O2, and O3) are shown in Supplementary Fig. 6a and b. When 50% of Co sites are replaced with Ni atoms, the vacancy formation energy can be significantly decreased, e.g., from 3.40 to 1.61 eV at O1 sites and from 3.49 to 1.72 eV at O2 sites (Supplementary Fig. 6c). The reduction of oxygen vacancy formation energies due to Ni doping has also been confirmed by our spin polarized DFT calculations (Supplementary Fig. 6d), in which PCO and PNC in antiferromagnetic (AFM) and ferromagnetic (FM) magnetic configurations are considered, respectively. Both AFM PCO and FM PNC are semiconducting with a band gap of 1.25 and 1.05 eV, respectively (Supplementary Fig. 7). Since the formation of oxygen vacancies is a prerequisite for water dissociative incorporation into the defective lattice, the formation energy reduction may induce essential water hydration, which is of high importance. The high-temperature in-situ X-ray diffraction turns out that the crystal structure evidently shows chemical expansion after water is introduced (Fig. 1c), indicating full hydration after 20 h at 600 °C (Supplementary Fig. 9). The hydration content is also characterized with thermogravimetric analysis (Supplementary Note 3). In Fig. 2a, water hydration behavior in PNC is compared with some recently developed oxygen electrode materials. Remarkably, PNC oxide exhibited a higher water insertion of 0.055% ± 0.003%, in contrast to a 0.035% ± 0.002% weight increase for PrBa_0.5_Sr_0.5_Co_1.5_Fe_0.5_O_5+δ_ (PBSCF) after hydrating at 500 °C for 15 h (Supplementary Fig. 10)^30,31^. PNC also showed higher hydration capability than conventional MIEC electrodes such as La_0.6_Sr_0.4_Co_0.2_Fe_0.8_O_3−δ_ (LSCF, ~0.031%) and PCO (~0.028%)_,_ which are normally rich in oxygen vacancies^32^. Clearly, the sufficient oxygen deficiency should not be the only factor considered when evaluating a proton conductor. Other factors, such as the energy of proton migration, may be taken in account in controlling the kinetics of the proton transport. Fig. 2d and e further shows the minimum energy path (MEP) calculation results along two representative proton transfer pathways via inter-octahedral hopping (Fig. 2b) and intra-octahedral hopping (Fig. 2c) in the bulk PNC, where a proton diffuses from one oxygen ion to a neighboring oxygen ion. For both cases, the proton migration energy in PNC is significantly reduced, suggesting that the Ni doping can accelerate proton transport through the bulk. The effects of magnetism on proton migration barriers are also shown in Supplementary Fig. 8. The two local minima in the MEPs for intra-octahedron proton hopping are the result of O–H bond tilting at the O3 site toward neighboring O2 sites, presumably due to hydrogen bonding. Consequently, the intra-octahedron proton migration path consists of three stages: (i) O2 to O3 jump, (ii) O–H bond rotation at O3 site from left to right, and (iii) O3 to O2 jump. The presence of protons in PNC was also confirmed by Fourier-transform infrared spectroscopy (FTIR) and temperature programmed desorption (TPD) (Supplementary Note 4). As temperature increased, the intensity of OH peak (3000–3500 cm^−1^) was continuously enhanced due to the loss of chemisorbed hydroxyl group at PNC surface at low temperature range (<400 °C) and proton defects in lattice at higher temperatures (Supplementary Fig. 11). The dehydrated water signal at temperature range (400~600 °C) was also found by mass spectroscopy, which corresponds to lattice hydroxyl in PNC (Supplementary Fig. 12). In addition, when the dense PNC membrane is used to permeate hydrogen, high fluxes of 4 × 10^−8^ and 1.5 × 10^−7^ mol cm^−2^ s^−1^ have been demonstrated at 500 °C in dry and wet 3% hydrogen respectively, indicating the co-existence of proton and electron conductivity (Fig. 2f). The hydrogen permeation rate is even higher than those of well-known cermet material Ni-BaCe_0.7_Zr_0.1_Y_0.1_Yb_0.1_O_3_ and mixed-conducting ceramic SrCe_0.95_Yb_0.05_O_3−δ_^33,34^. It should be noted that fully replacing Co with Ni can cause decomposition into Pr_2_NiO_4+δ_ and NiO in air above 1000 °C, which limits full Ni substitution for maximizing proton conductivity^35^. The chemical stability in reducing condition after the experiment was confirmed by XRD (Supplementary Note 5 and Supplementary Fig. 13).

### Electrochemical performances of PCECs with PNC oxygen electrode

**Fig. 3:**
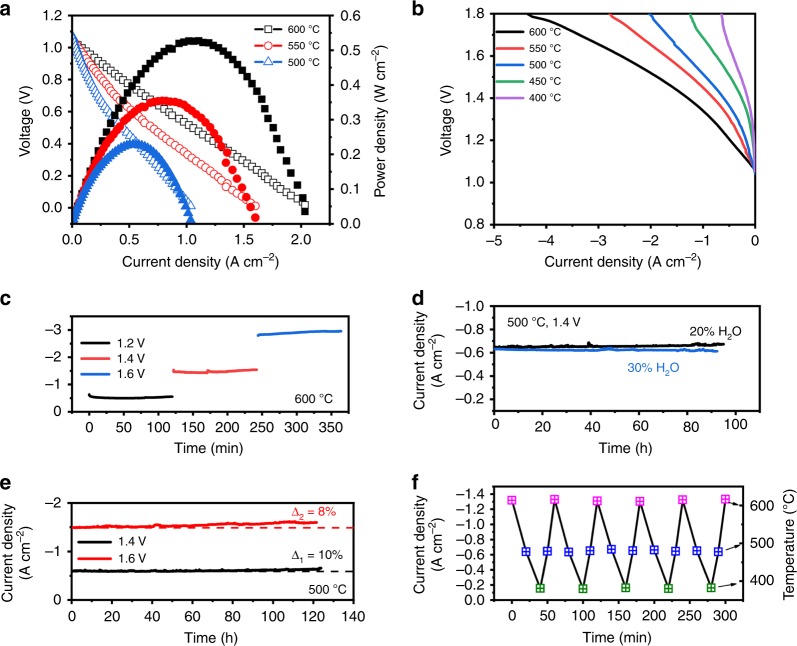
Electrochemical performances of PCEC with PNC oxygen electrode. **a** Performance in fuel cell mode (pure H_2_ is used as fuel). **b** Current–voltage curves measured in electrolysis mode at various temperatures and dry 10% H_2_ and wet air (~10% H_2_O) are used as reactant gas in hydrogen electrode and oxygen electrode, respectively. **c** Current density response at different constant voltage of 1.2, 1.4 and 1.6 V at 600 °C. **d** Durability testing of the cell in different steam concentrations (20 and 30% H_2_O) at 500 °C. **e** Stability testing of the cell at constant voltage of 1.4 V and 1.6 V at 500 °C. **f** Thermal cycle durability of the cell between 400 and 600 °C.

Figure 3a shows the electrochemical performance of the proton conducting solid oxide fuel cell (H-SOFC) with a PNC electrode and BaCe_0.4_Zr_0.4_Y_0.1_Yb_0.1_O_3_ (BCZYYb4411) electrolyte operating at 400~600 °C in fuel cell mode with pure hydrogen as fuel. The high open circuit voltages (OCVs) suggest the presence of a dense electrolyte membrane and good sealing, e.g., 1.06 V at 600 °C, which is close to the theoretical Nernst potential of 1.13 V. The peak power densities are 528 mW cm^−2^ at 600 °C, 354 mW cm^−2^ at 550 °C, and 230 mW cm^−2^ at 500 °C, respectively, which are among the highest performances of H-SOFCs. For example, the performance in the present work at 600 °C is nearly three times higher than the cell with a hybrid catalyst modified (La_0.6_Sr_0.4_)_0.95_Co_0.2_Fe_0.8_O_3−δ_ cathode (198 mW cm^−2^ at 600 °C) and 147% higher than for a cell with optimized electrolyte and electrode materials (214 mW cm^−2^ at 600 °C)^36,37^. The results are attributed to the excellent ORR activity for PNC electrode even without microstructure optimization, indicated from impedance spectra (Supplementary Note 6 and Supplementary Fig. 14a). When the cell was switched to electrolysis mode with humidified air (~10% H_2_O) in the PNC electrode and 10%H_2_/90%Ar in the Ni-BCZYYb4411 electrode, the OCVs were slightly decreased due to the diluted hydrogen, e.g. from 1.09 V in 97% H_2_ to 1.04 V in 10% H_2_ (Fig. 3b). At the applied voltage of 1.4 V, the electrolysis current densities of 1.31, 0.82, and 0.62 A cm^−2^ were achieved at 600 °C, 550 °C and 500 °C respectively. As the operating temperature was further decreased to 450 °C and 400 °C, the electrolysis current densities presented values of 0.27 A cm^−2^ at 450 °C and 0.15 A cm^−2^ at 400 °C at 1.4 V, which are among the best performances reported at such low temperatures to our best knowledge. These promising results suggest that PNC electrode is active towards ORR and WOR at low temperatures. As indicated from the impedance spectra, the electrolyte resistances dominate the total cell resistance, which is related to the conductivity and thickness of the electrolyte. The interfacial polarization resistances, largely contributed by the electrode at a bias of 1.4 V are 0.025, 0.075 and 0.130 Ω cm^2^ at 600 °C, 550 °C and 500 °C, respectively. At 450 °C and 400 °C, the resistance increases to 0.35 Ω cm^2^ and 0.6 Ω cm^2^ (Supplementary Fig. 14b). The effect of gas condition at oxygen electrode and hydrogen electrode sides on the cell performance was also investigated (Supplementary Note 7). When the wet air was switched to wet oxygen, the cell showed higher current density; however, it exhibited poor performance in argon (Supplementary Fig. 15). The results are consistent with the finding in the study of symmetric cell, in which higher oxygen partial pressure can improve electrode polarization resistance at OCV conditions (Supplementary Fig. 16). On the hydrogen electrode side, the less concentrated hydrogen gas may enhance the hydrogen production, and the cell showed the highest current density in pure argon. The electrolysis characteristics of the cell with PNC electrode at different voltages (1.2~1.6 V) are shown in Fig. 3c. The cell showed slight activation at each voltage, which may result from the decrease of interfacial electrode polarization. To examine long-term stability, the cell was operated at 1.4 V and 1.6 V, each for 120 h (Fig. 3e). As a result, the current density increased by 10% and 8% respectively after the entire electrolysis processes, which may be attributed to the improved electrode/electrolyte interface as indicated by the impedance spectra (Supplementary Note 8 and Supplementary Fig. 17). The performance stability in higher steam concentrations (20 and 30%) was also examined (Fig. 3d). The current density was relatively stable, and minor changes were caused by the slight fluctuation of steam supply, further indicating the PNC’s good chemical stability and interfacial binding with the electrolyte in high-content steam. The little dependence of current density on steam concentration may be attributed to the cell configuration with very thin oxygen electrode layer and small active area, in which the mass transport should not be the rate-limiting step as expected as the gas can be regarded as fully saturated. The lower oxygen partial pressure by increased steam concentration may affect electrode conductivity that leads to negative effect on electrolysis performance. The high-performance stability can be attributed to chemical stability of PNC electrode and strong interfacial bonding between electrode and electrolyte, which is indicated from the systematic stability study on material structure and symmetric cell under different water pressure (Supplementary Fig. 18). Another longer-term stability testing was performed in 20% H_2_O at 500 °C for 480 h, showing the robust stability (Supplementary Fig. 19). The thermal cycling capability of the cell was evaluated by measuring the current density at 1.4 V as the temperature changed periodically (Fig. 3f). After five cycles between 400 °C and 600 °C, the cell performance remained identical, indicating a good thermal cycling durability in a benchtop test. The Faradaic efficiencies at different temperatures were measured with larger electrolysis cells (1 inch in diameter), e.g., 79.3% at 1.32 V and 600 °C, 83.4% at 1.3 V and 550 °C, and 86.2% at 1.3 V and 500 °C (Supplementary Fig. 20). As steam pressure increased from 15 to 50%, the efficiency was improved slightly due to increased proton concentration (Supplementary Note 9).

### Enhanced performances by optimizing microstructure of triple conducting electrode

**Fig. 4:**
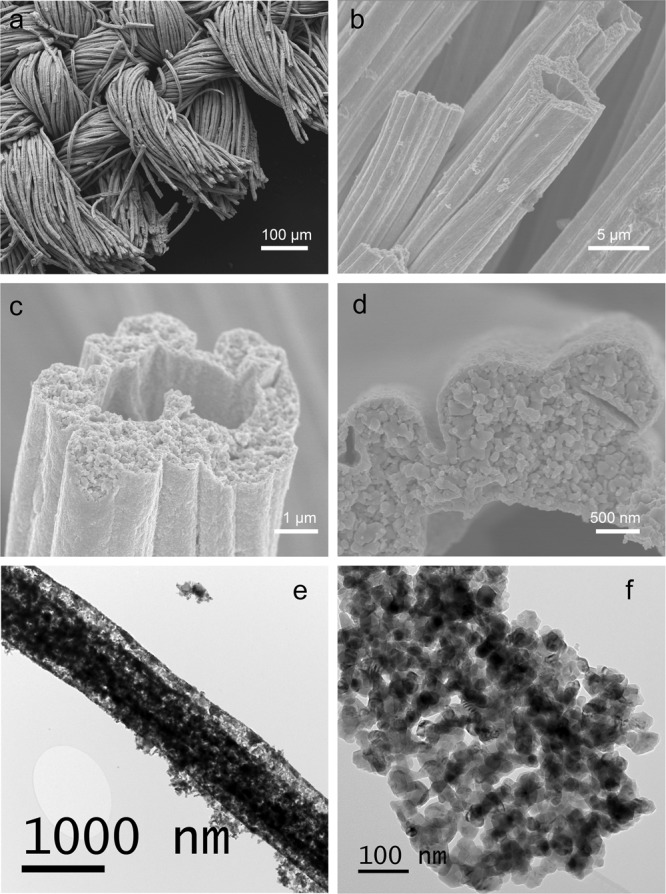
Nanofiber-structured PNC mesh with high porosity and active nanoparticles. **a**–**d** Scanning electron microscopy images of the electrode mesh with different magnification showing hollow-fiber-like string self-architectured to form mesh structure. **e**, **f** Transmission electron microscopy images of a single nanofiber composed of ~50 nm particles.

**Fig. 5:**
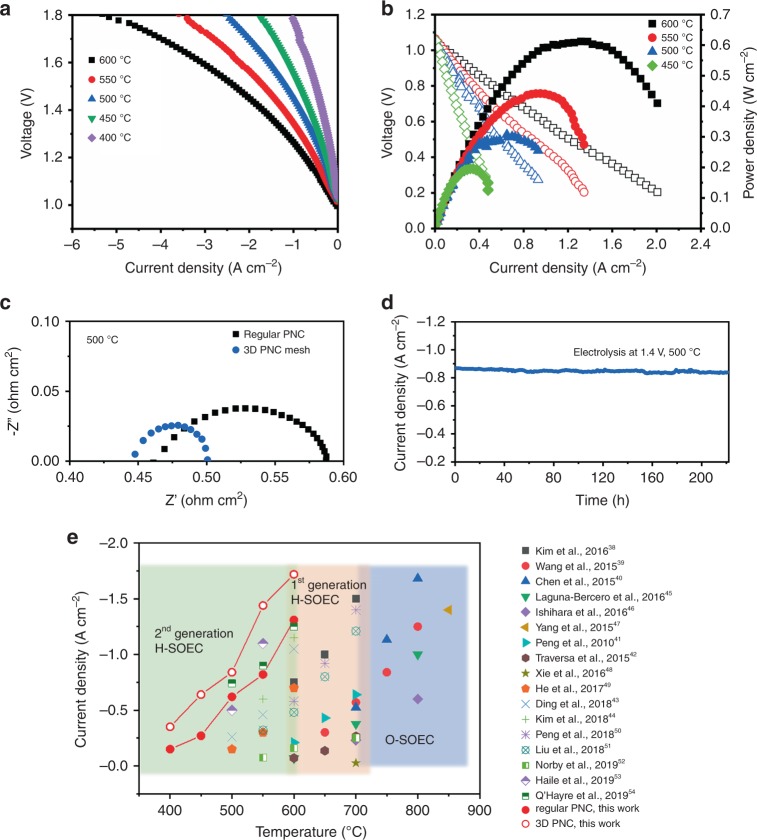
Enhanced electrochemical performances with nanofiber-structured electrode. **a** Current–voltage curves measured in electrolysis mode at various temperatures and dry 10% H_2_ and wet air (~10% H_2_O) are used as reactant gas in hydrogen electrode and oxygen electrode, respectively. **b** Performance in fuel cell mode (pure H_2_ is used as fuel). **c** Comparison of impedance spectra under 1.4 V at 500 °C for cells with regular or 3D PNC electrode. **d** Durability testing of the cell in electrolysis mode at 500 °C. **e** Performance comparison of representative SOECs at electrolysis voltage of 1.4 V for oxide-ion conducting SOEC (O-SOEC), first-generation proton conducting SOEC (H-SOEC) operating at high temperatures (600~700 °C), second-generation H-SOEC at intermediate temperatures (<600 °C) and high-performing electrolysis cell developed in this work.

The electrochemical performance of this triple conducting PNC electrode enabled PCEC can be further improved by optimizing the microstructure to improve gas diffusion throughout the entire electrode (Supplementary Note 10). A self-architectured mesh-like electrode is synthesized to construct a highly porous frame for enhanced mass transport (Fig. 4). Each bundle consists of hollow PNC fibers with throughout holes (~3 µm in diameter) for facilitating gas diffusion to entire nanoparticles-structured surface where reactions occur. For each single fiber, it is composed of nanoparticles with size ranging from 20 to 50 nm. When this nanostructured electrode is incorporated into the cell (Supplementary Fig. 21), the better performance is obtained. As shown in Fig. 5a, the electrolysis current density at each temperature has been increased, e.g., 1.18 A cm^−2^ at 1.3 V and 1.72 A cm^−2^ at 1.4 V, comparing with 0.85 and 1.31 A cm^−2^ for regular PNC at 600 °C. In fuel cell mode, the peak power density was also correspondingly improved. At 600 °C, 611 mW cm^−2^ was achieved with the increase of 15.7% (Fig. 5b). The performance enhancement mainly results from the decrease of electrode polarization resistance. For example, the electrode polarization resistance was reduced from 0.13 to 0.055 Ω cm^2^ at 500 °C (Fig. 5c). The results are attributed to the improved concentration polarization resistance and electrode reaction kinetics at interface due to high porosity and fine nanoparticles. The durability of the cell with this nanostructured electrode was examined. At 1.4 V and 500 °C, the electrolysis current density didn’t show observable degradation over 220 h while the improvement on the mesh-electrode/electrolyte interfacial strength is needed to further enhance the long-term stability (Fig. 5d).

The performance of this PCEC ranks at the top among recent developed SOECs operated at high and intermediate temperature ranges (Supplementary Note 11). The electrolysis performances obtained in this work were compared with those results from oxygen-ion conducting SOECs (O-SOEC) and proton conducting SOECs (H-SOEC), as shown in Fig. 5e (and Supplementary Table 1)^38–54^. The first-generation H-SOECs have shown the potential of reducing the operating temperature down to intermediate temperature (600~700 °C) with improved performance. For example, Kim et al. reported a highly efficient electrolysis cell using layered perovskite PrBaMn_2_O_5+δ_ and PBSCF as both electrodes with current density of 0.75 A cm^−2^ at 1.4 V and 600  °C, representing the best performance up to date for O-SOECs^38^. In contrast, H-SOEC shows the capability of decreasing operating temperature down to 500~600 °C while still maintaining comparable performance. For example, Peng and Liu et al. recently reported H-SOECs with new electrodes exhibiting remarkable performances (0.34 A cm^−2^ at 1.4 V and 600 °C for Pr_2_NiO_4+δ_ electrode, and 0.58 A cm^−2^ at the same condition for SrEu_2_Fe_1.8_Co_0.2_O_7−δ_ electrode)^50,51^. By further optimizing electrode composition of NdBa_0.5_Sr_0.5_Co_1.5_Fe_0.5_O_5+δ_ layered perovskite and adopting proper operating condition, electrolysis current density can be improved to 0.6 A cm^−2^ at 1.4 V at a lower temperature of 550 °C^44^. Most recently there have been several H-SOECs developed with high electrolysis performances at reduced temperature, such as 0.5 A cm^−2^ at 500 °C reported by Haile^53^ and 0.74 A cm^−2^ at 1.4 V in O’Hayre’s work^54^. The PNC electrode in this study clearly demonstrates one of the best performances, with highest current density at 500~600 °C (1.72 A cm^−2^ at 600 °C, 1.44 A cm^−2^ at 550 °C, 0.84 A cm^−2^ at 500 °C, under 1.4 V). The cell also obtained reasonable performance at reduced operating temperatures, e.g., 0.64 A cm^−2^ at 450 °C, and 0.35 A cm^−2^ at 400 °C, which shows the potential of PCECs operating at even lower temperatures than second-generation SOECs.

### Self-sustainable reversible operation of PCEC

**Fig. 6:**
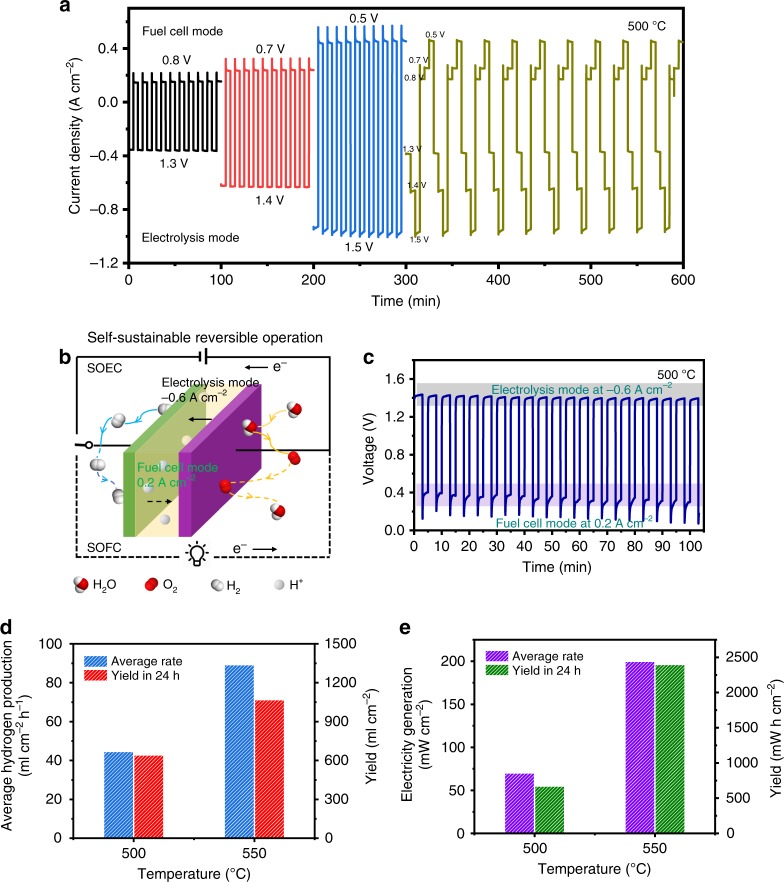
Reversible operation and analysis in hydrogen and power generation. The cycling reversible operation of the electrochemical cell between electrolysis mode (hydrogen production) and fuel cell mode (electricity generation) at different current densities to examine the capability of converting hydrogen generated by electrolysis into electricity. **a** Reversible operation with external hydrogen supply: current density responses as working mode transiently changing between electrolysis and fuel cell at 500 °C. **b** Schematic of self-sustainable reversible operation without external hydrogen feeding. **c** Voltage observation under switchable electrolysis and fuel cell current densities at 500 °C. **d** Average hydrogen production based on results on current density and Faradaic efficiency and hydrogen yield assuming the cell is operated for 24 h. **e** Average electricity generation rate and electricity yield in 24 h.

To demonstrate self-sustainable reversible operation between water electrolysis and electricity generation, the electrochemical cell with regular PNC electrode was examined in a series of cycling experiments. First, the cell was operated at different voltages to switch working mode between SOEC and SOFC, e.g., 1.3, 1.4, and 1.5 V for producing hydrogen and 0.8, 0.7, and 0.5 V for generating electricity (Fig. 6a). The current densities at each mode are stable with the trend of slight improvement^55^. The continuous hydrogen and power generation at different voltages in ten cycles demonstrates stable operation with resulting current densities. Furthermore, the capability of self-sustainable reversible operation was demonstrated after terminating additional hydrogen supply. As shown in Fig. 6b, the cell was firstly operated at an electrolysis mode with a constant current density of 0.6 A cm^−2^ for three minutes to generate hydrogen, which was instantly consumed by switching to a fuel cell mode at 0.2 A cm^−2^ for two minutes. The stable electrolysis voltages in each cycle suggest that the reversible operation did not induce negative effect on the cell performance (Fig. 6c). In the electricity generation process, the gradual deterioration of cell performance with cycles may be caused by the fact that hydrogen generated in the electrolysis mode cannot be completely used in the chamber, which is under further optimization on testing fixture. The successful demonstration of such electrolysis/fuel-cell mode cycling without additional hydrogen at hydrogen electrode is the first time to achieve the energy storage functionality in PCECs by self-sustainably converting electricity and hydrogen from each other. When the temperature was increased to 550 °C, the cell cycling under larger current densities showed the similar behavior but more stable fuel-cell process (Supplementary Note 12 and Supplementary Fig. 22). The hydrogen production rate and electricity generation yield are calculated from current density, active electrode area and Faradaic efficiency to determine daily expected outputs in hydrogen and electricity in this reversible cell (Fig. 6d and e). The average hydrogen production rate is 44.7 ml cm^−2^ h^−1^ at 500 °C and 89.3 ml cm^−2^ h^−1^ at 550 °C, respectively, when Faradaic efficiency is considered. The expected daily production yield is 643 ml cm^−2^ and 1069 ml cm^−2^ at 500 °C and 550  °C respectively, based on current electrolysis/fuel-cell cycling scheduling. When the electrolysis hydrogen is used as fuel to convert back to electricity, the cell can deliver power with an average rate of 70  mWh cm^−2^ at 500 °C and 200 mWh cm^−2^ at 550 °C, indicating the probability of better performance when the experiment apparatus is improved. As calculated, the daily generated electricity is 671 mWh cm^−2^ and 2400 mWh cm^−2^ at 500 °C and 550 °C, respectively. Therefore, the results fully demonstrate the capability of this PCEC on reversibility operation to store and convert hydrogen fuel transiently with stable electrode/electrolyte interface observed after testing (Supplementary Note 13 and Supplementary Fig. 23).

## Discussion

Developing a triple (H^+^/O^2−^/eʹ) conducting oxide as oxygen electrode for PCECs is technically critical for facilitating catalytic activity towards water oxidation and ORRs at reduced temperatures. Based on perovskite structure, the flexibility on crystal distortion allows element replacement to tailor the atomic arrangement to possibly increase the chance of material hydration and proton migration. However, the direct evidences on characterizing the existence of proton diffusion for new materials are rarely available in the development. In this work, hydration is considered as the prerequisite of proton conductivity. The high-temperature X-ray diffraction directly observes the chemical expansion behavior due to insertion of water molecule into atomic lattice to form the proton defects. The thermogravimetric analysis result can also give partial confirmation of weight increase attributing to proton formation in the water-containing atmosphere. Quantifying the proton/electron mixed conductivity is implemented by hydrogen permeation rate measurement when the feeding gas is changed from dry to wet condition. The high flux rate of 1.5 × 10^−7^ mol cm^–2^ s at 500 °C in wet 3% H_2_ has surpassed single-phase mixed conducting ceramic SrCe_0.95_Yb_0.05_O_3−δ_ at 900 °C by 17 times and Ni-BaCe_0.7_Zr_0.1_Y_0.1_Yb_0.1_O_3_ cermet membrane at 700 °C by 25%, which demonstrates higher proton conductivity of PNC oxide than these conventional barium/strontium cerate based proton conductors. The originated proton conduction in PNC can be attributed to lower proton migration energies validated by DFT study.

The triple conduction in PNC has facilitated the catalytic activity significantly and consequently promoted the electrochemical performance in both electrolysis and fuel cell modes. While the water splitting and oxygen reduction occur at the entire electrode surface, the improvement of mass transport is needed to make gas diffusion throughout electrode grains more necessary. The incorporation of the three-dimensional mesh-like PNC electrode can make full use of the high porosity and active nanoparticles of nanofibers for superior performance. Our recent result has demonstrated that hydrogen production rate has been greatly improved by this self-architectured ultra porous layered perovskite PBSCF steam electrode^43^, e.g., 0.85 A cm^−2^ at 1.3 V and 600 °C which was increased from regular electrode (0.55 A cm^−2^). This ultra-porous structure could be further optimized by changing the treatment temperature to make a compromise between porosity and active sites.

The high effectiveness of PCECs is necessary to convert a high fraction of electrons and water into hydrogen. The Faradaic efficiencies of electrolzyers based on proton conducting electrolytes have been recently reported by several research groups^52–54^. Electronic leakage within the electrolyte under direct current voltage becomes severe with increasing temperature and oxygen partial pressure, which can reduce the efficiency. On the other hand, the p-type electronic charge carrier concentration can be decreased by introducing higher steam pressure that can shift the equilibrium towards higher proton concentration, thus a higher proton transfer number. Haile et al. reported 74~84% efficiency for realistic electrolysis operational conditions in which the steam concentration was low (3% H_2_O). With increased water pressure, the efficiency has been improved to a reasonable range (75~90%). Therefore, lower operating temperature and electrolysis voltage are beneficial to electron-to-hydrogen conversion efficiency, which further explains the importance of active oxygen electrode that can facilitate second-generation H-SOECs towards operation in reduced temperatures. In addition, the further decrease of Ce content may also improve the efficiency while the performance and stability are maintained.

In summary, a TCO electrode of PNC has been developed as an oxygen electrode for proton conducting electrochemical cells with superior performances at intermediate temperature range, resulting in a self-sustainable reversible operation demonstrated by generating electricity with hydrogen as the fuel transiently produced in electrolysis process. The DFT calculation and experimental characterizations have confirmed the formation of proton defects and reduction of proton migration energy, which facilitate the extension of active WOR/ORRs throughout the entire electrode. Rational design of PNC electrode could be a guideline for other electrochemical systems; and the self-sustainable PCEC represents a novel prototype of energy storage and conversion with hydrogen as an energy carrier.

## Methods

### Materials synthesis and characterizations

The PNC electrode powder was synthesized by modified glycine-citrate combustion method, where nitric acid and glycine were used as parallel complexing agents. The precursor solution was prepared by dissolving stoichiometric amounts of Pr(NO_3_)_3_·6H_2_O (99.9%, Alfa Aesar), Ni(NO_3_)_3_·6H_2_O (99+%, Alfa Aesar), and Co(NO_3_)_3_·6H_2_O (99%, STREM Chemicals) in citrate-glycine aqueous solution. Under heating and stirring conditions, the viscous gel ignited to obtain the ash that was calcined at 1000 °C in air for 5 h to form perovskite structure. The crystal structure, crystal lattice, and element valence states were examined by X-ray diffraction (XRD, Rigaku SmartLab), transmission electron microscopy (TEM, JEOL 4000 EX) and by X-ray photoelectron spectroscopy respectively. The oxygen non-stoichiometry was measured by iodometric titration method and thermogravimetric analysis. The hydration behavior of PNC and several other conventional electrode materials (LSCF and PBSCF) in humid air was studied by thermogravimetric analysis (TA Instruments, Q500). The weight change of the powder sample (~50 mg) was recorded when the temperature was ramped to 950 °C with a 1 °C min^−1^ ramping rate and dwelled for 1 h. After cooling to 500 °C, the gas condition was switched from dry air to wet air (~3% H_2_O) and hydration process lasted for about 15 h. The high-temperature X-ray diffraction was carried out by examining the phase structure at 600 °C when the atmosphere exposed to the sample was changed from dry air to wet air. Fourier-transform infrared spectroscopy (FTIR, Nicolet iS50) was used to investigate the water and hydroxyl groups bound to the surface of PNC as function of temperature. The powder sample was hydrated at 600 °C and then cooled down to room temperature. After flushing with dry argon for 1 h, the baseline was collected. The in-situ measurement was carried out every 50 °C from 50 to 600 °C in dry argon with ramping rate of 10 °C min^−1^. Temperature programmed desorption (TPD, OmniStar Analysis system) was utilized to study the dehydration process in PNC as powder sample was heated to 850 °C with dry oxygen flushing the quartz chamber.

### Hydrogen permeation

Permeation measurement was performed with disk-shaped PNC dense membrane (diameter of 12 mm). The sample were prepared by dry pressing the powder into cylindrical die to form the green pellets followed by sintering at 1250 °C for 8 h in air. The sample (~0.3 mm in thickness) was polished and painted with Pt paste for quick firing at 800 °C to remove the carbon. Then the pellet was sealed in the house-made double chamber reactor using Ceramabond 552 and glass powder. After curing and glass sealing process, the mechanical leakage calibration was carried out and the result was used for calculating permeation flux. Argon was used as sweep gas (50 ml min^−1^) on the permeation side whereas a mixture of dry or wet 3%H_2_ (1%N_2_, 96%He, 100 ml min^−1^) was fed to the hydrogen-rich chamber. The gas chromatography (Shimadzu 2010 plus) was used to monitor the change of hydrogen concentration at sweep side. The permeation flux rate was calculated by the average of three measurement results.

### PCEC fabrication and electrochemical measurements

The hydrogen electrode supported half cells of NiO-BCZYYb4411-electrode/BCZYYb4411-electrolyte (~1 inch in diameter) were fabricated by laminating the green tapes prepared by tape-casting method, following by sintering at 1450 °C in air for 8 h. The PNC-electrode slurry was screen painted onto electrolyte to form the full PCECs with active area about 3.1 cm^2^ after firing at 950 °C for 5 h. The mesh-like ultra-porous PNC electrode was synthesized by template method, which was calcined at 800 °C to form the phase structure, as described in elsewhere^43^. The 3D PNC mesh was adhered onto electrolyte by applying very small amount of PNC slurry for good bonding. The final PCEC cell was sealed in the house-made reactor using Ceramabond 552 and glass powder with the steam electrode side up exposing to humid air (80 ml min^−1^) and hydrogen electrode side (30 ml min^−1^) towards the inner tube for analysis of exhaust gas. The electrochemical performances of current–voltage characteristic curves, impedance spectra, electrolysis stability at a constant voltage/current–density were collected by electrochemical working station (Solartron 1400). Faradaic efficiencies were measured based on the ratio between the experimental hydrogen generation rate and theoretical one at fixed current densities. Gas chromatography was used to monitor hydrogen concentration at open circuit condition and constant electrolysis current density for calculating actual hydrogen generation amount. At each point, at least three measurements were executed for average values. The volume/mass variation of exhaust gas was also considered for accurate calculation. The thermal cycling was carried out with heating rate 5 °C  min^−1^ and cooling rate 10 °C min^−1^ respectively while the current density was recorded at a fixed electrolysis voltage of 1.4 V. The post-test samples were examined by scanning electron microscope (SEM, FEI Quanta 250 FEG) to observe the layers and interfaces. For element mapping in transmission electron microscope (JEOL JEM-2010 FASTEM), the sample was prepared with focused ion beam technique (Thermo Fisher, Strata 400).

### Computational method

DFT calculations are performed using the Vienna ab initio Simulation Package (VASP) code^56^. The electron-ion interactions are described by the projector-augmented wave (PAW) method^57^. For exchange-correlation functional, we employ the generalized gradient approximation of Perdew, Burke, and Ernzerhof (PBE-GGA)^58^, augmented by a Hubbard correction on the 3*d* orbitals of cobalt and nickel to account for the localization and strong correlations of 3*d* electrons. A simplified rotationally invariant approach to GGA + U by Dudarev et al. is employed^59^, where the only input parameter is U-J. The U-J values for Co and Ni are respectively set at 3.3 and 6.4 eV according to a previous study by Wang et al.^60^. The cutoff energy for plane-wave basis sets is set at 520 eV. A large 160-atom supercell, which is a 2 × 2 × 1 extension of the 40-atom cell, is used to minimize the unphysical electronic and elastic interactions between a defect and its periodic images. For Brillouin zone sampling, a 2 × 2 × 4 Monkhorst-Pack k-point mesh is used. The migration barrier for proton hopping between neighboring oxygen atoms are calculated by a climbing image nudged elastic band (CI-NEB) technique^61^. To model non-charge-neutral defects such as proton, we artificially add/subtract electrons from the system, with the charges being compensated by a neutralizing jellium background.

A Co^3+^ ion in an octahedral environment such as PCO and PNC can have three possible electron spin states: low-spin with no unpaired electrons (*S* = 0), intermediate-spin (*S* = 1), and high-spin (*S* = 2). At low temperatures, the ground state of PCO is nonmagnetic with Co^3+^ in low-spin configuration^62^, while the intermediate-spin or high-spin states can be activated at finite temperatures. However, the precise picture of the temperature-dependent spin state of Co^3+^ is still not clear.

## Supplementary information

Supplementary Information

## Data Availability

The data supporting the findings of this study are available within the article and Supplementary Information or from the authors upon request.
