# Supplementary material for: Self-sustainable protonic ceramic electrochemical cells using a triple conducting electrode for hydrogen and power production
Source: Nat Commun. 2020 Apr 20;11:1907. doi: 10.1038/s41467-020-15677-z (PMC7171140; doi:10.1038/s41467-020-15677-z)
Supplement: Supplementary file 1 — Supplementary Information [file 41467_2020_15677_MOESM1_ESM.pdf]

Supplementary Materials for

Self-Sustainable Protonic Ceramic Electrochemical Cells Using A Triple Conducting Electrode for Hydrogen and Power Production

Hanping Ding, Wei Wu, Chao Jiang, Yong Ding, Wenjuan Bian, Lucun Wang, Boxun Hu, Prabhakar Singh, Christopher J. Orme, Yunya Zhang, Dong Ding*

*Corresponding author. E-mail: dong.ding@inl.gov

Supplementary Figure 1. Material analysis.
Supplementary Figure 2. XRD Rietveld refinement.
Supplementary Figure 3. Oxygen content of PNC material at different temperatures.
Supplementary Figure 4. The XRD examination of chemical compatibility between PNC electrode and BCZYYb electrolyte after calcining the mixture powder (50:50 wt%) at 1000 °C for 10 h in air.
Supplementary Figure 5. X-ray photoelectron spectroscopy (XPS) analysis on PNC powders.
Supplementary Figure 6. DFT calculation on formation energy of extrinsic oxygen vacancy.
Supplementary Figure 7. Electronic density of states (DOS).
Supplementary Figure 8. Minimum energy paths for intra-octahedron hopping of proton in AFM PCO and FM PNC.
Supplementary Figure 9. In-situ high-temperature XRD in hydration process at 600 °C.
Supplementary Figure 10. The TGA curves for different electrode materials (LSCF, PBSCF and PNC) to study the hydration process at 500 °C in wet air.
Supplementary Figure 11. In-situ FTIR spectra for PNC in dry argon at different temperatures (50~600 °C).
Supplementary Figure 12. H_2O-TPD profiles of the PNC with the region for adsorbed water and proton defect when the sample was heated to 850 °C in flushed dry oxygen.
Supplementary Figure 13. The chemical stability of PNC in reducing condition.
Supplementary Figure 14. Electrochemical impedance spectra of the cell at different conditions.
Supplementary Figure 15. The performance of the electrochemical cell operating in both fuel cell and electrolysis modes at 500 °C with electrodes exposing to different gas conditions.
Supplementary Figure 16. The effect of (A) oxygen partial pressure and (B) applied voltage on electrode polarization characterization in a symmetric cell with PNC as electrode at 500 °C.
Supplementary Figure 17. The impedance spectra of the cell before and after the long-term testing measured under a voltage bias of 1.4 V at 500 °C.
Supplementary Figure 18. Long-term stability of material structure and electrode activity.
Supplementary Figure 19. Long-term stability of PCEC under 1.4 V and 20% H_2O at 500 °C for 480 h.
Supplementary Figure 20. Faradaic efficiency of BCZYYb4411-based PCEC.
Supplementary Figure 21. Cross-sectional view of the post-test cell with PNC mesh-like electrode.
Supplementary Figure 22. Reversible operation of the electrochemical cell between electrolysis and fuel cell modes at elevated temperature of 550 °C.

Supplementary Figure 23. Microstructure characterization of post-test PCEC with PNC oxygen electrode.
Supplementary Table 1. Comparisons of electrolysis current density at different testing conditions for high-temperature and intermediate-temperature oxide-ion conducting or proton conducting electrolyzers.
Supplementary Note 1: Characterization of PNC electrode.
Supplementary Note 2: DFT calculations
Supplementary Note 3: Hydration behavior of PNC oxide observed in high-temperature X-ray diffraction and thermogravimetry analysis.
Supplementary Note 4: Fourier-transform infrared spectroscopy (FTIR) and temperature programmed desorption (TPD) techniques for detecting proton defects in PNC.
Supplementary Note 5: Chemical stability in hydrogen permeation experiment and reducing condition.
Supplementary Note 6: Electrochemical impedance spectra at reduced temperatures (400~600 °C).
Supplementary Note 7: Hydrogen production in different conditions.
Supplementary Note 8: Long-term stability of material structure and electrode activity.
Supplementary Note 9: Faradaic efficiency in BCZYYb4411-based PCEC at different operating conditions.
Supplementary Note 10: Incorporation of 3D mesh-like PNC electrode into PCEC.
Supplementary Note 11: Performance comparisons.
Supplementary Note 12: Self-sustainable reversible operation between electrolysis mode and fuel cell mode.
Supplementary Note 13: Microscopy characterization of the PCEC after test.
Supplementary References

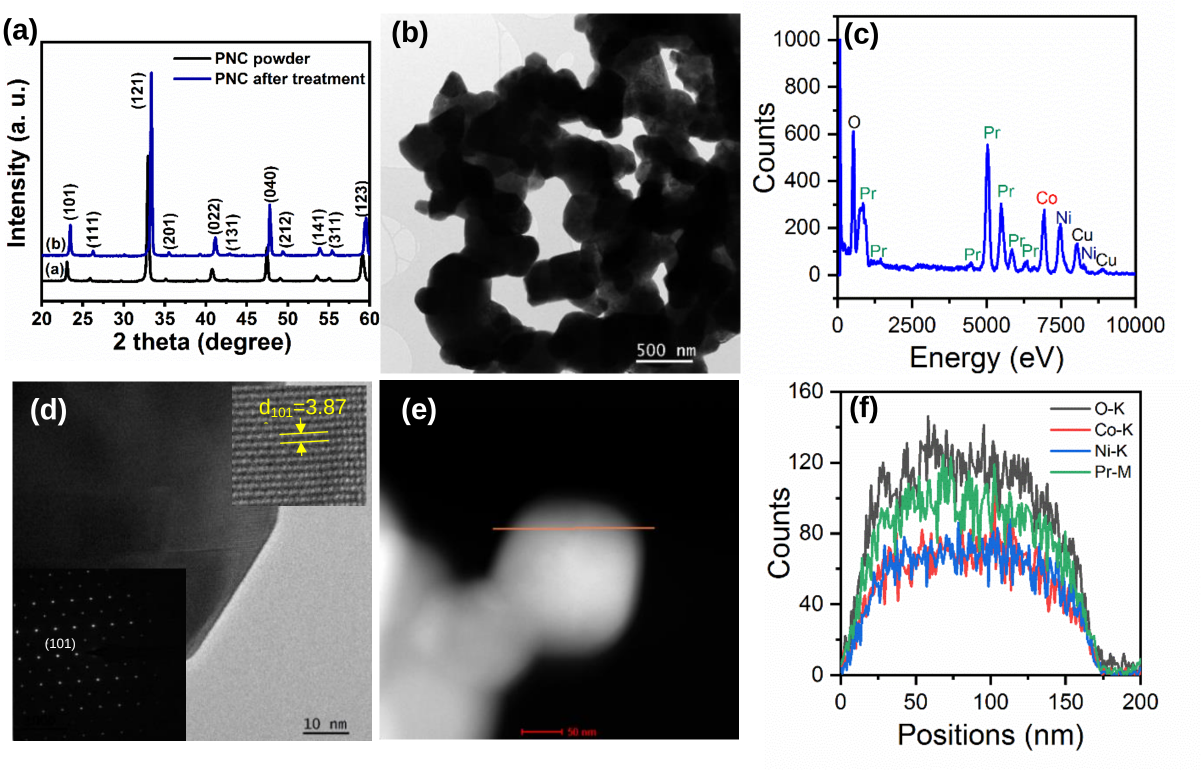

Supplementary Figure 1. Material analysis. (a) XRD patterns of as-prepared PNC powders and post-treated sample after exposing in 50%H_2O at 600 $^{\circ}$C for 100 hours. (b) Bright-field TEM image of powder morphology. (c) Energy dispersive X-ray (EDX) spectroscopy analysis. (d) High-resolution TEM lattice fringe image (inserts are enlarged lattice and selected area electron diffraction SAED pattern). (e) TEM image of particle tip. (f) Element line-scan along the direction on the tip in (e).

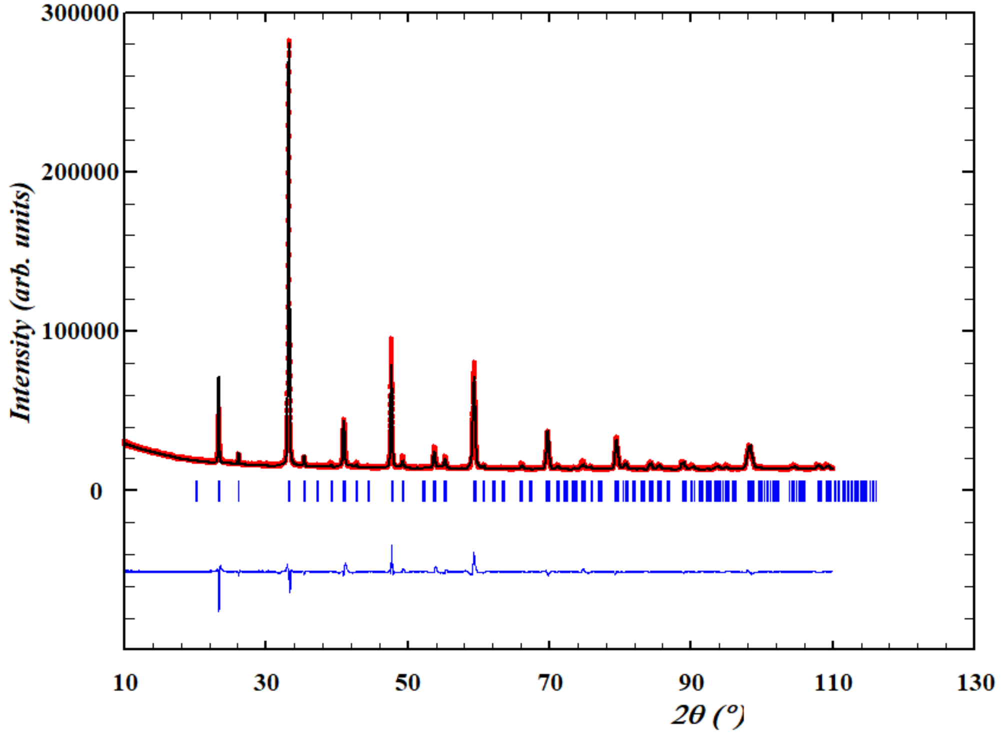

Supplementary Figure 2. XRD Rietveld refinement. Experimental (○, red), calculated and difference (solid lines, black and blue) XRD pattern of PNC: indexed to an orthorhombic *Pbnm* space group with unit cell: a=5.405 Å, b=5.380 Å, and c=7.617 Å. The vertical bars show the Bragg angle positions corresponding to the structure.

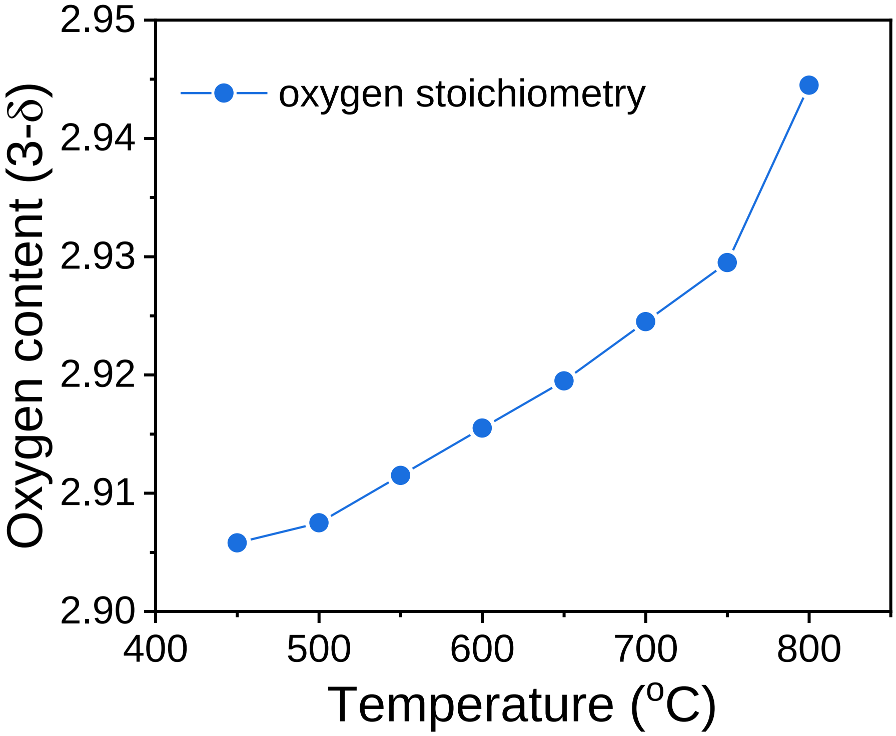

Supplementary Figure 3. Oxygen content of PNC material at different temperatures (450~800 °C) as obtained from the TGA and titration results.

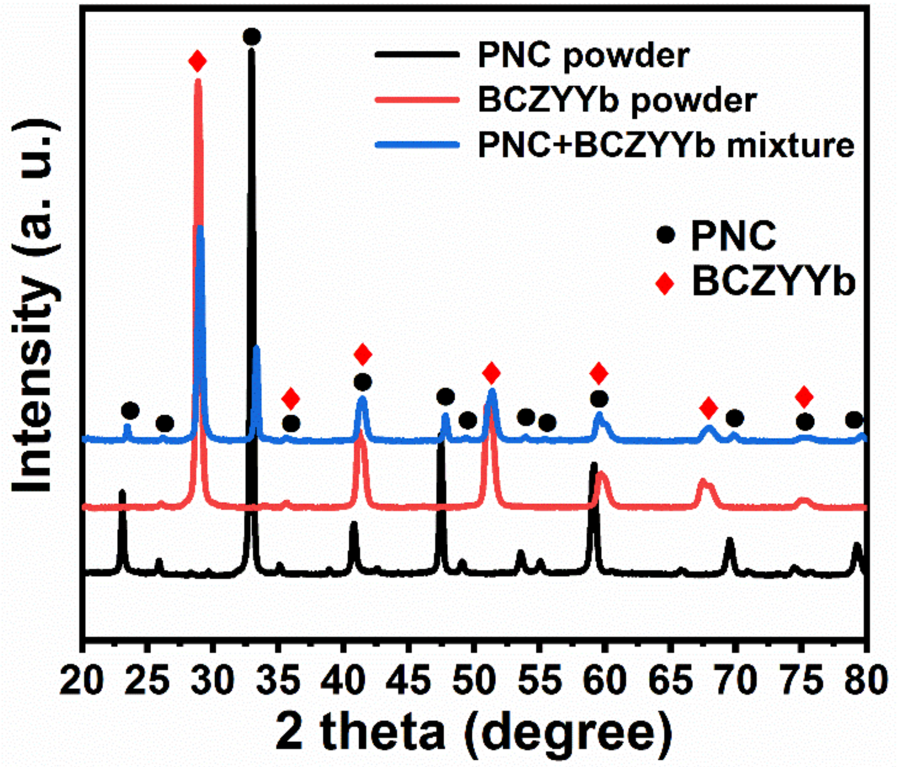

Supplementary Figure 4. The XRD examination of chemical compatibility between PNC electrode and BCZYYb electrolyte after calcining the mixture powder (50:50 wt%) at 1000 °C for 10 h in air.

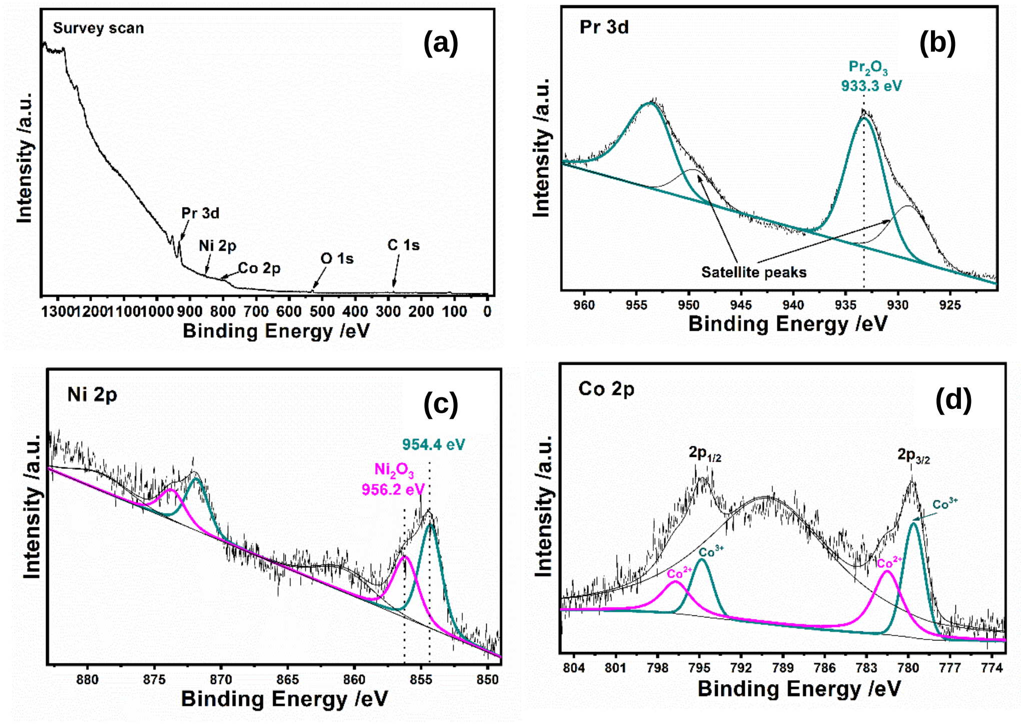

Supplementary Figure 5. X-ray photoelectron spectroscopy (XPS) analysis on PNC powders. (a) Survey scan. (b) Pr 3d. (c) Ni 2P. (d) Co 2p.

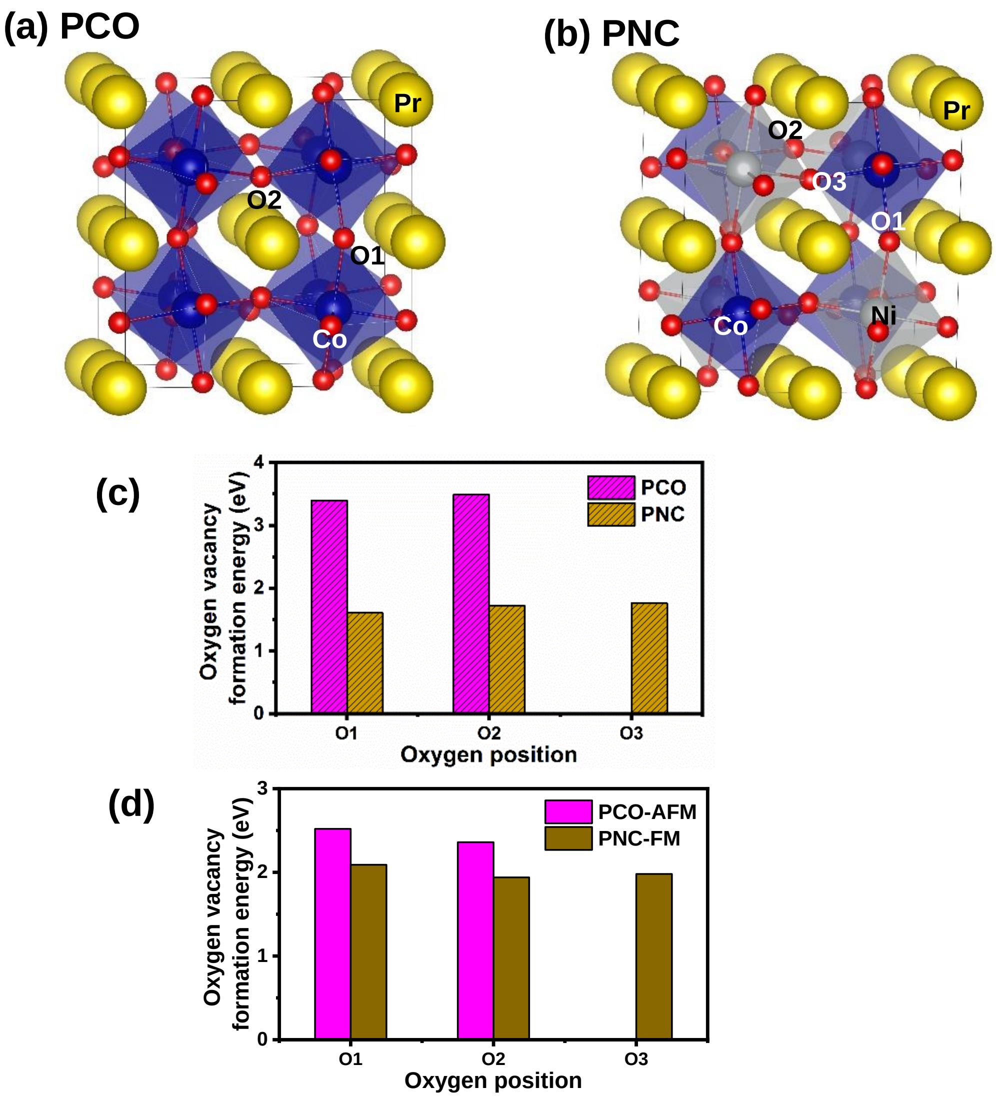

Supplementary Figure 6. DFT calculation on formation energy of extrinsic oxygen vacancy. (a-b) 160-atom supercell structure of PCO and PNC compounds. (c) Formation energies based on GGA+U modeling at different lattice oxygen positions (O1 and O2 for PCO; O1, O2, O3 for PNC). (d) Formation energies for AFM PCO and FM PNC.

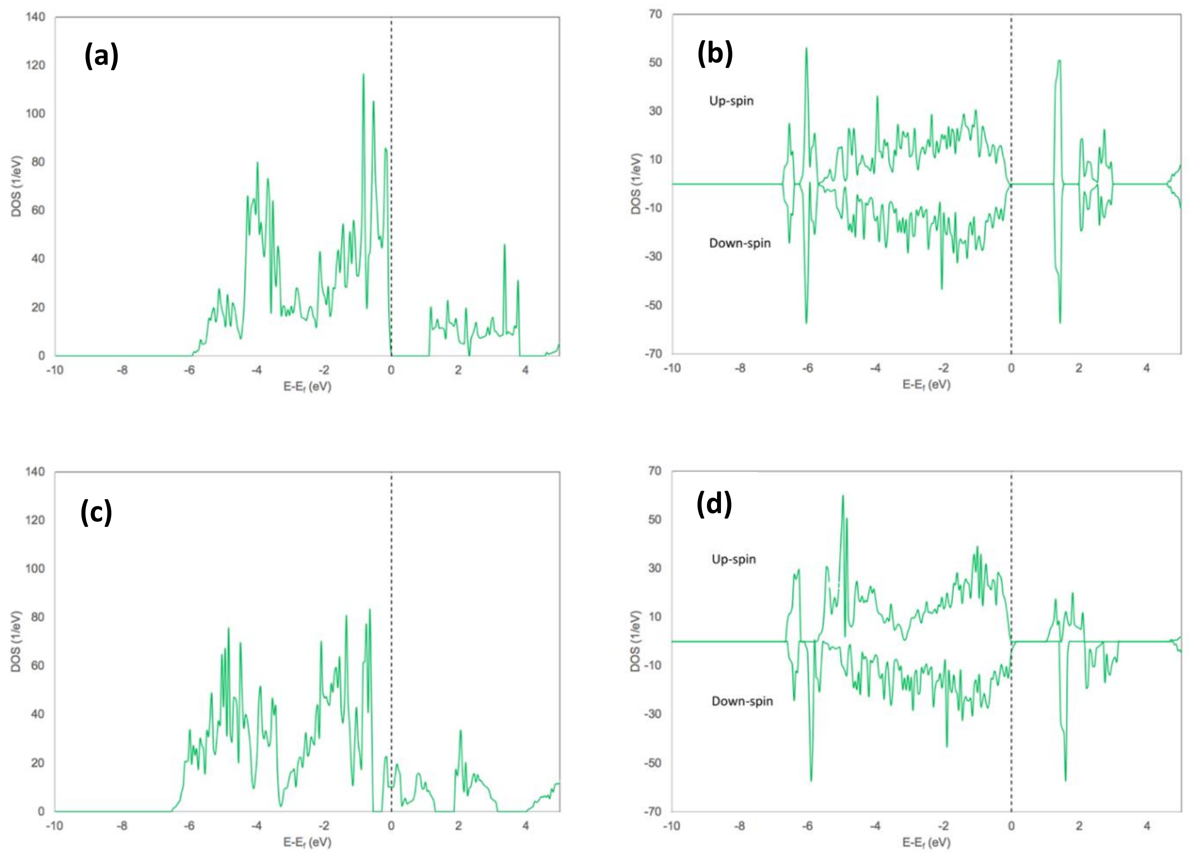

Supplementary Figure 7. Electronic density of states (DOS). (a) Non-magnetic PCO. (b) AFM PCO. (c) Non-magnetic PNC. (d) FM PNC. The vertical lines denote the Fermi level.

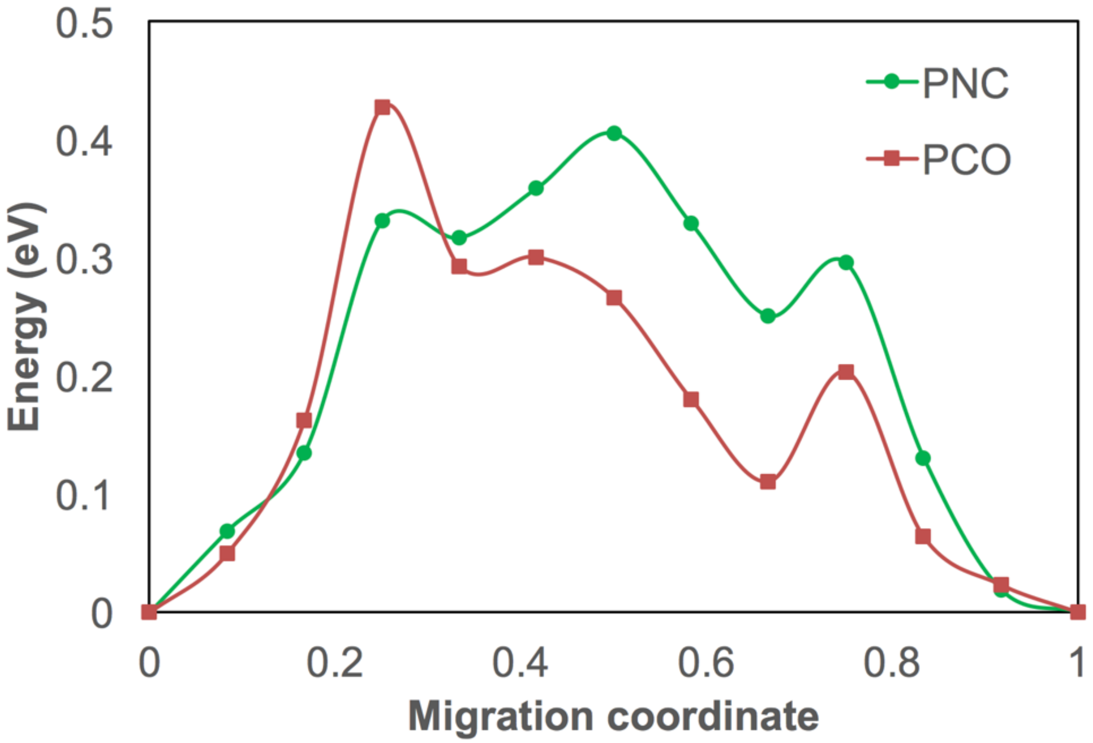

Supplementary Figure 8. Minimum energy paths for intra-octahedron hopping of proton in AFM PCO and FM PNC.

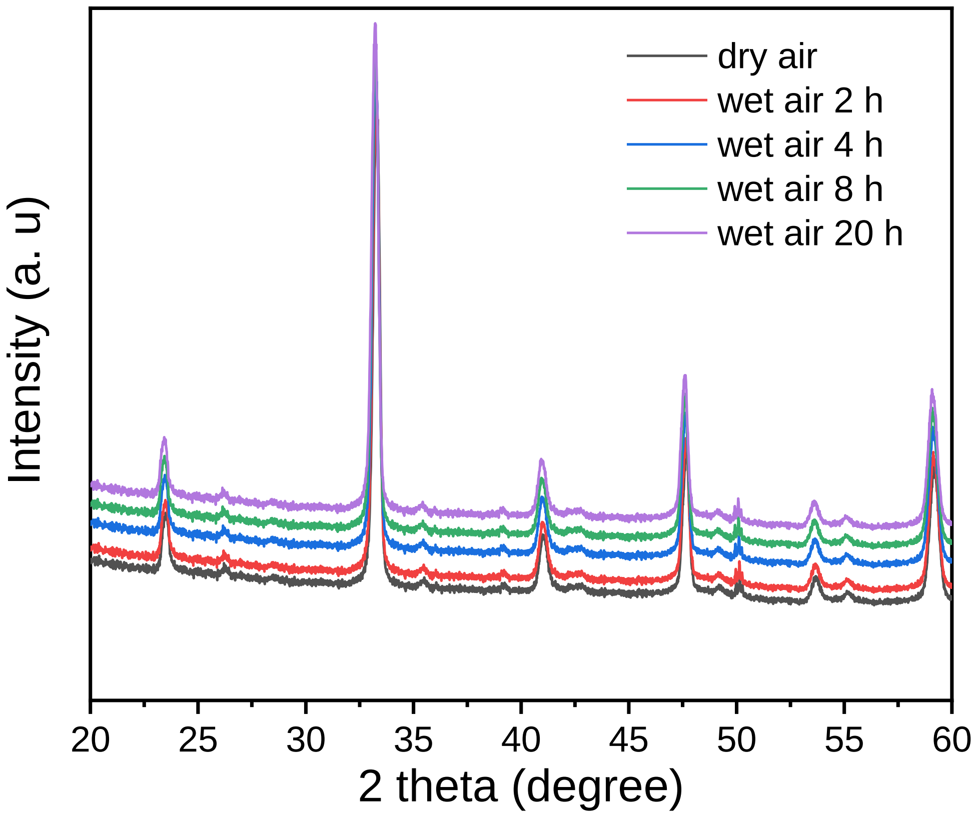

Supplementary Figure 9. In-situ high-temperature XRD in hydration process at 600 °C. The chemical expansion is observed by switching the exposed gas from dry air to wet air.

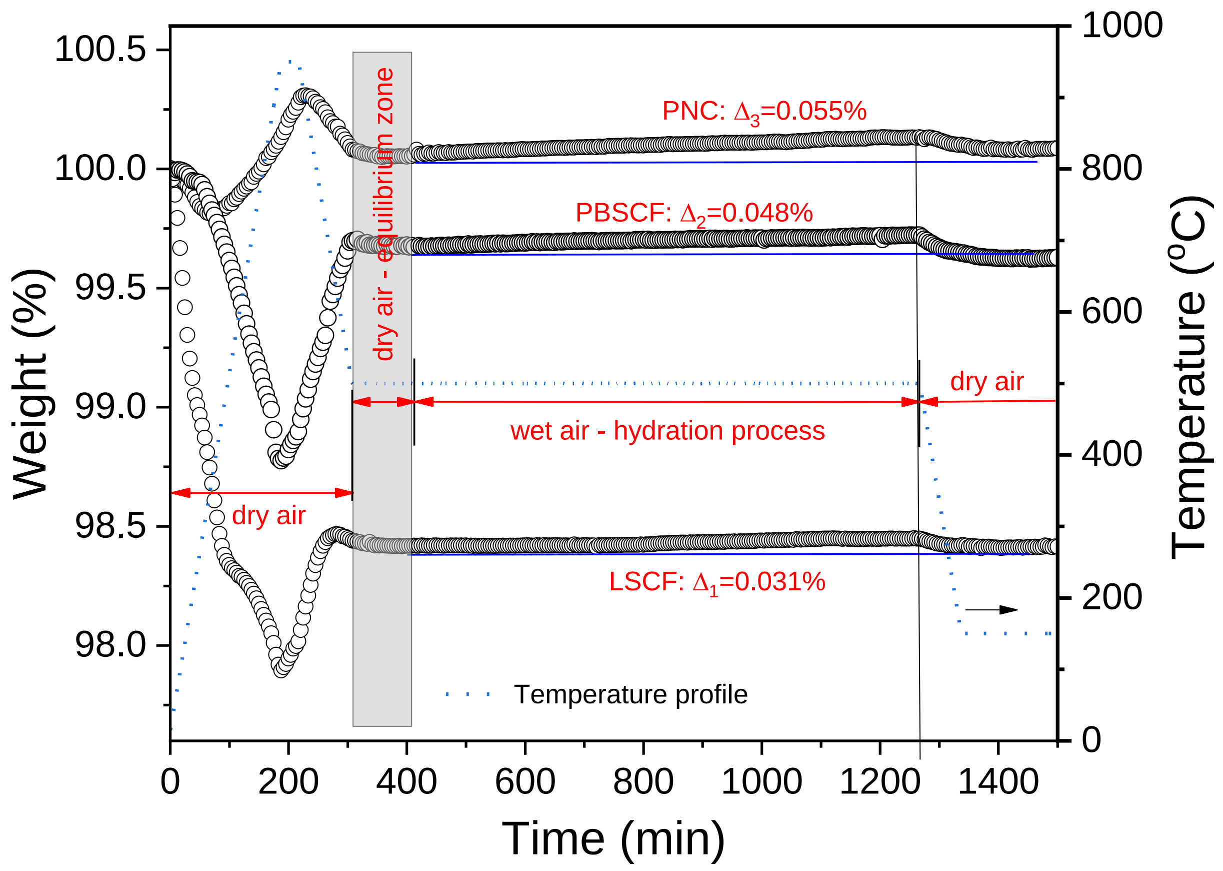

Supplementary Figure 10. The TGA curves for different electrode materials (LSCF, PBSCF and PNC) to study the hydration process at 500 °C in wet air.

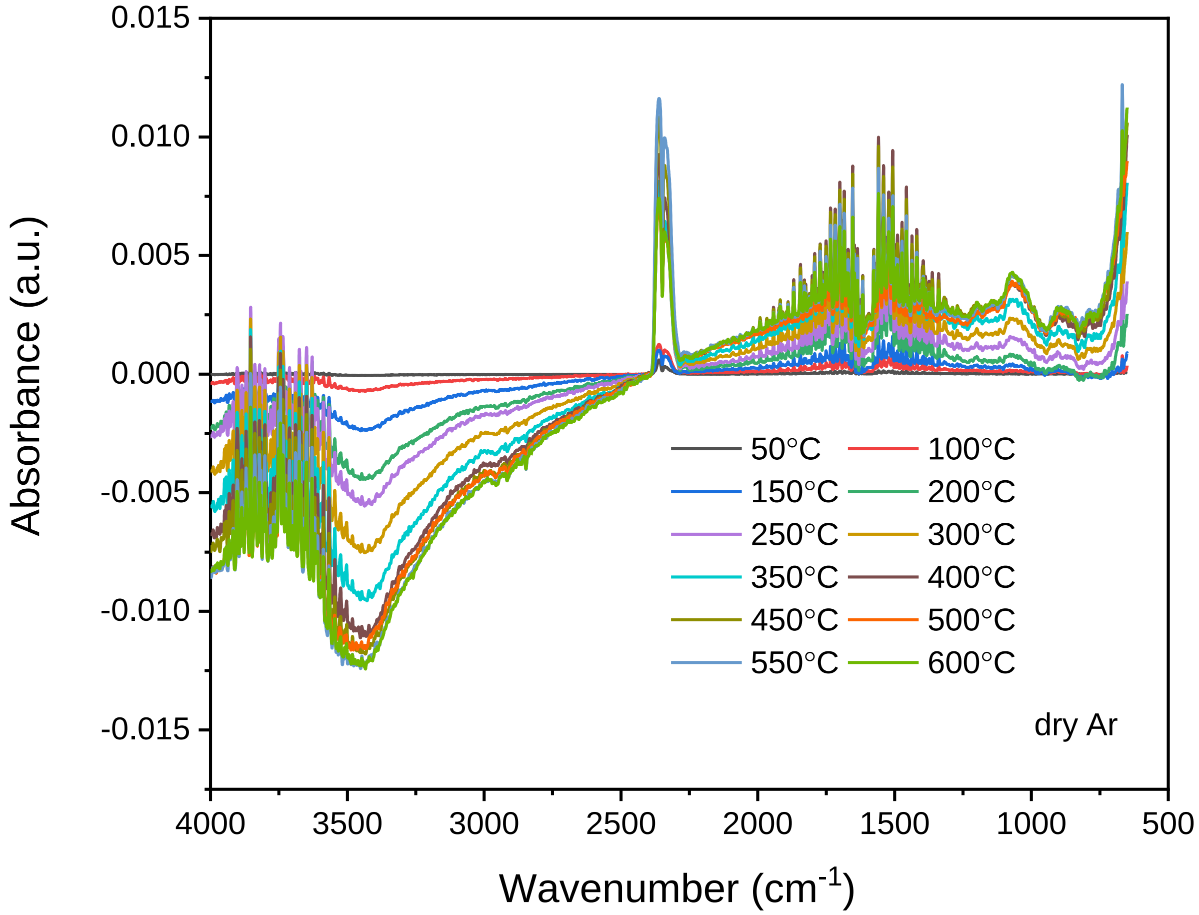

Supplementary Figure 11. In-situ FTIR spectra for PNC in dry argon at different temperatures (50~600 °C).

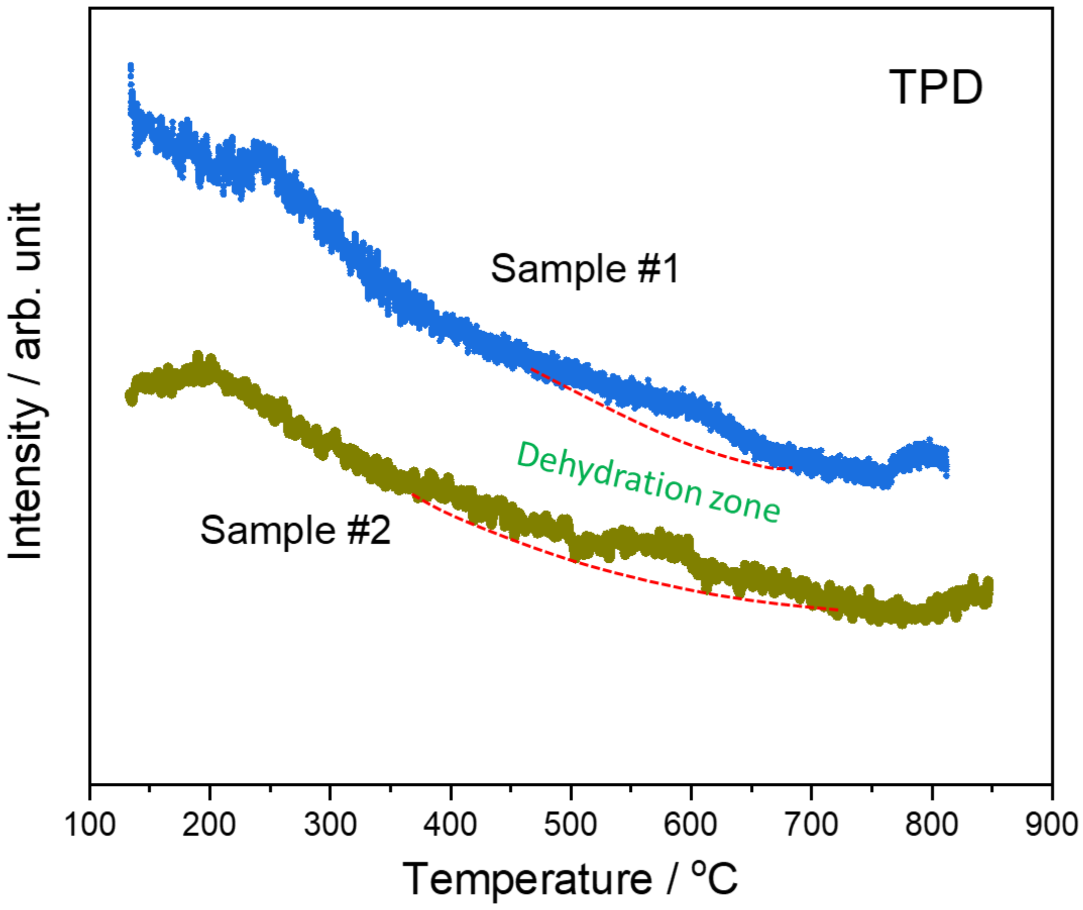

Supplementary Figure 12. H_2O-TPD profiles of the PNC with the region for adsorbed water and proton defect when the sample was heated to 850 °C in flushed dry oxygen.

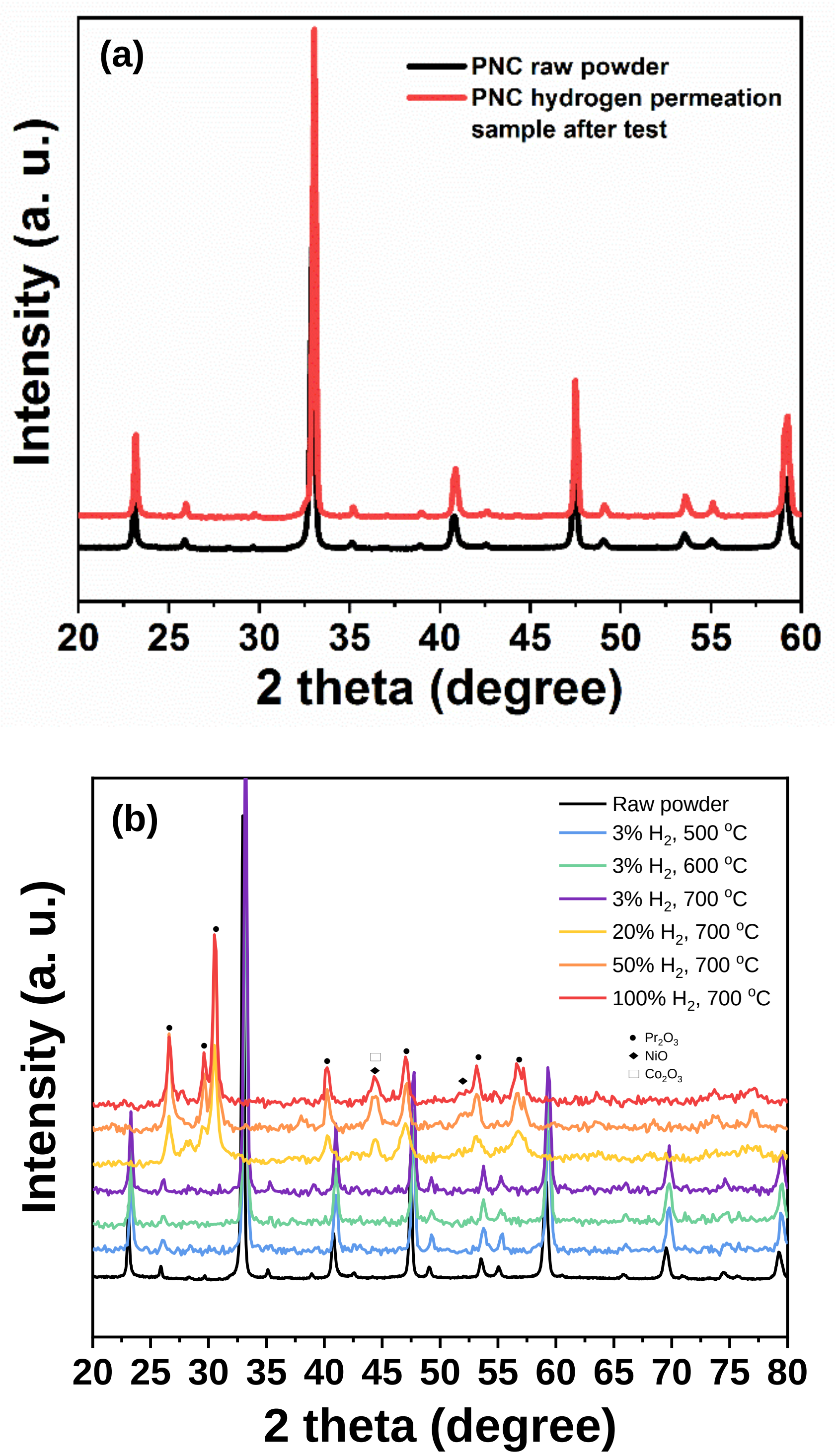

Supplementary Figure 13. The chemical stability of PNC in reducing condition. (a) XRD pattern comparison of PNC powder and pellet sample after hydrogen permeation experiment. (b) Examination of PNC powder in more reducing condition and higher temperatures.

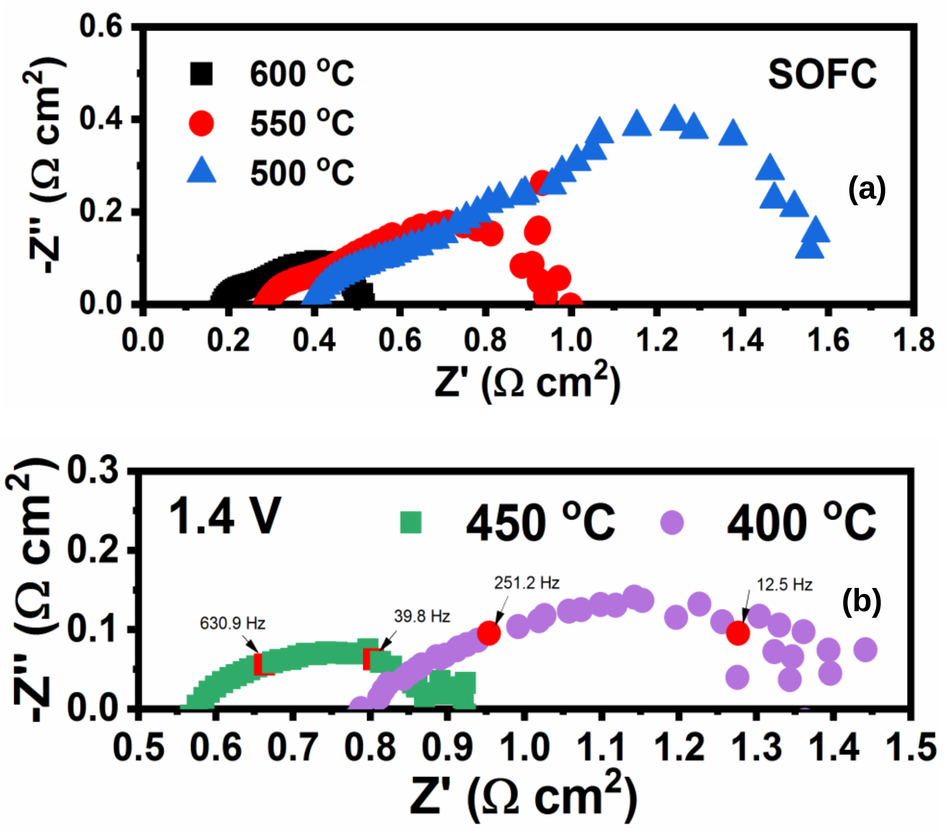

Supplementary Figure 14. Electrochemical impedance spectra of the cell at different conditions. (a) Fuel cell mode at open circuit conditions at 500~600 °C. (b) Electrolysis mode with applied voltage of 1.4 V at 450 and 400 °C.

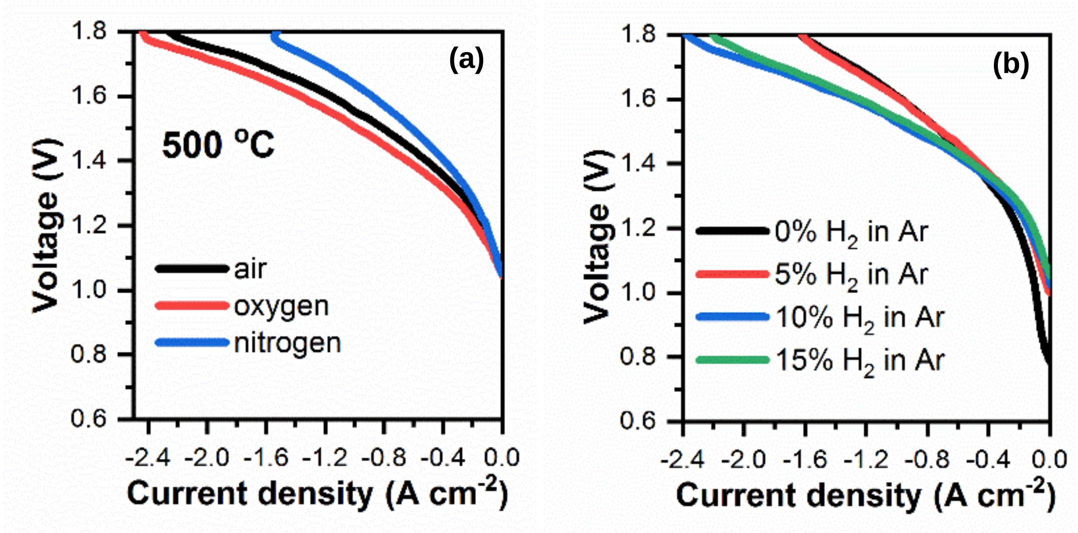

Supplementary Figure 15. The performance of the electrochemical cell operating in both fuel cell and electrolysis modes at 500 °C with electrodes exposing to different gas conditions. (a) Steam electrode: nitrogen, air or oxygen; hydrogen electrode: 10% H_2. (b) Hydrogen electrode: H_2 concentration from 0% to 15%; steam electrode: wet air.

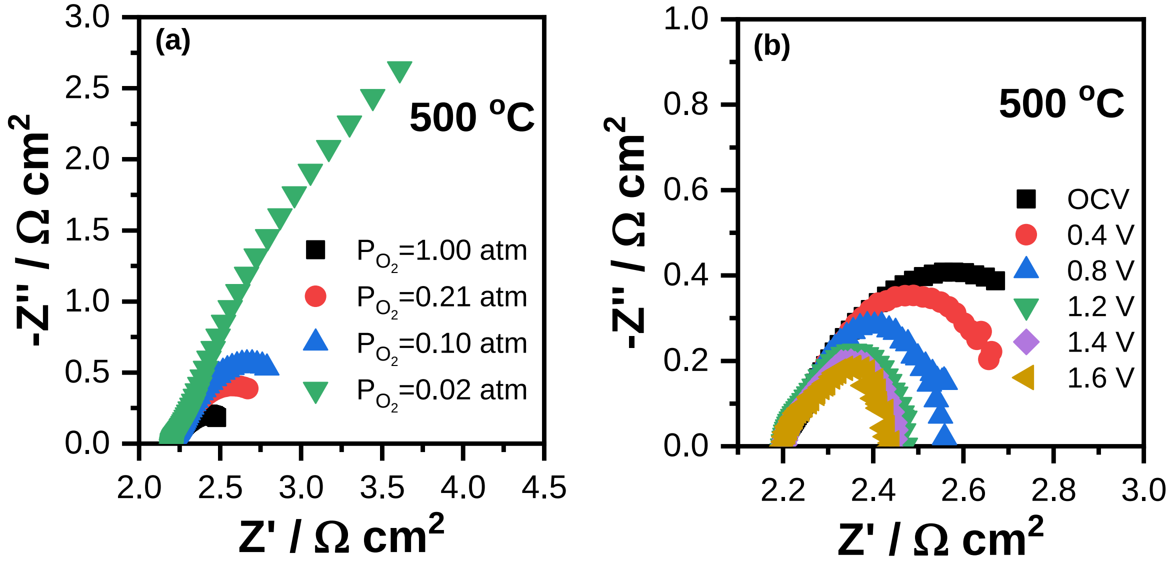

Supplementary Figure 16. The effect of (a) oxygen partial pressure and (b) applied voltage on electrode polarization characterization in a symmetric cell with PNC as electrode at 500 °C.

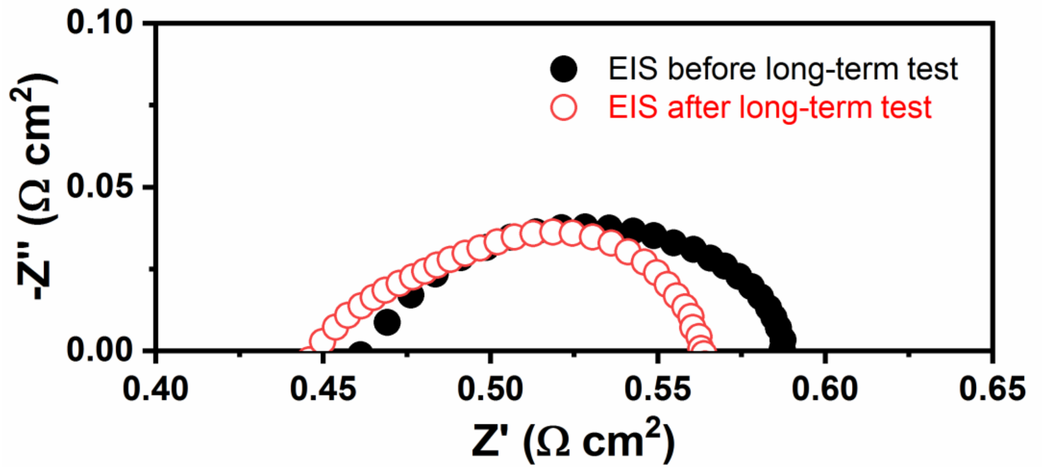

Supplementary Figure 17. The impedance spectra of the cell before and after the long-term testing measured under a voltage bias of 1.4 V at 500 °C.

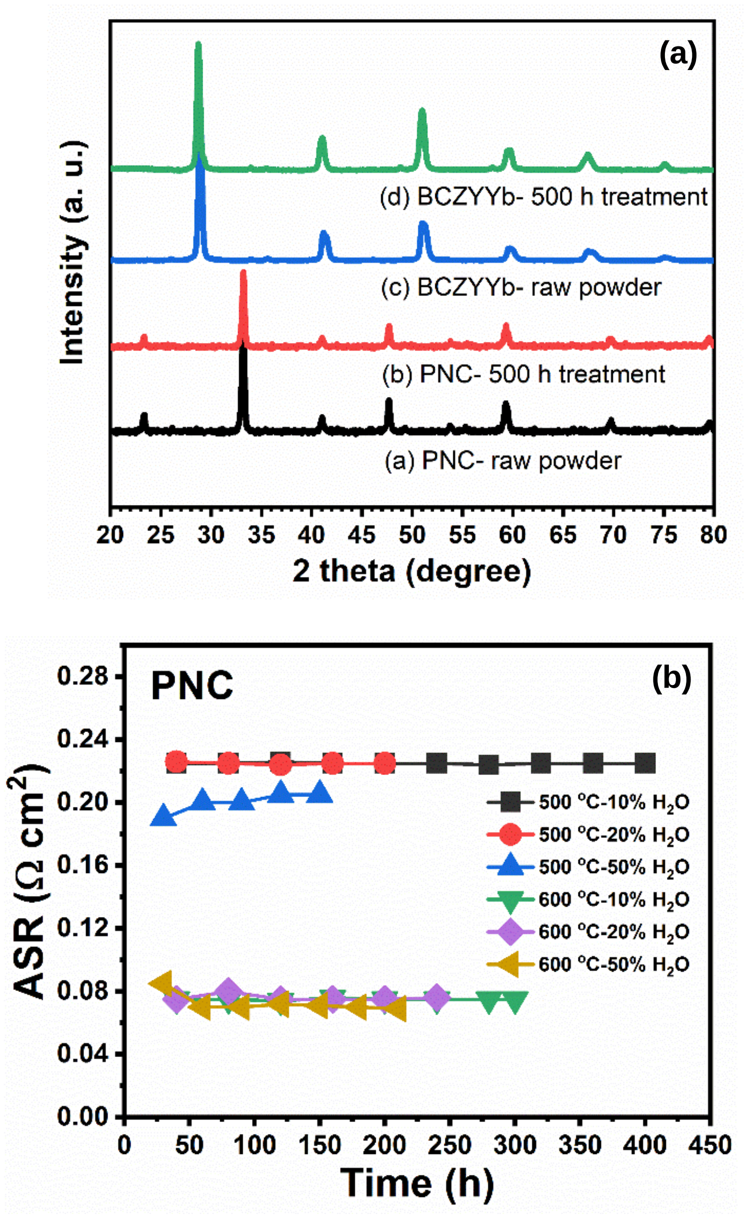

Supplementary Figure 18. Long-term stability of material structure and electrode activity. (a) crystal structure of BCZYYb electrolyte and PNC electrode after treated in 20% H_2O. (b) Area specific resistances (ASRs) of PNC electrode in symmetric cells at different temperature (500 and 600 °C) and water pressure (10%, 20% and 50%).

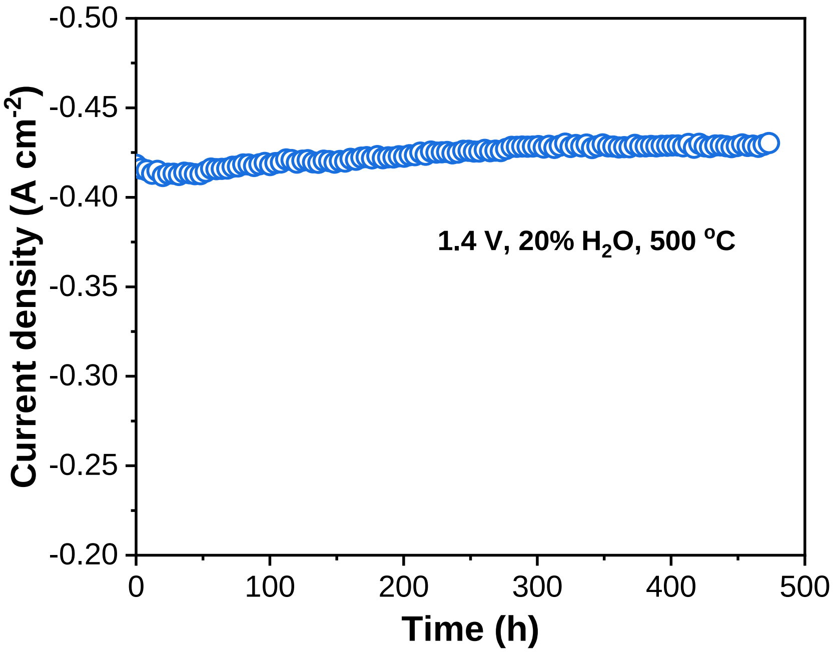

Supplementary Figure 19. Long-term stability of PCEC under 1.4 V and 20% H_2O at 500 °C for 480 h.

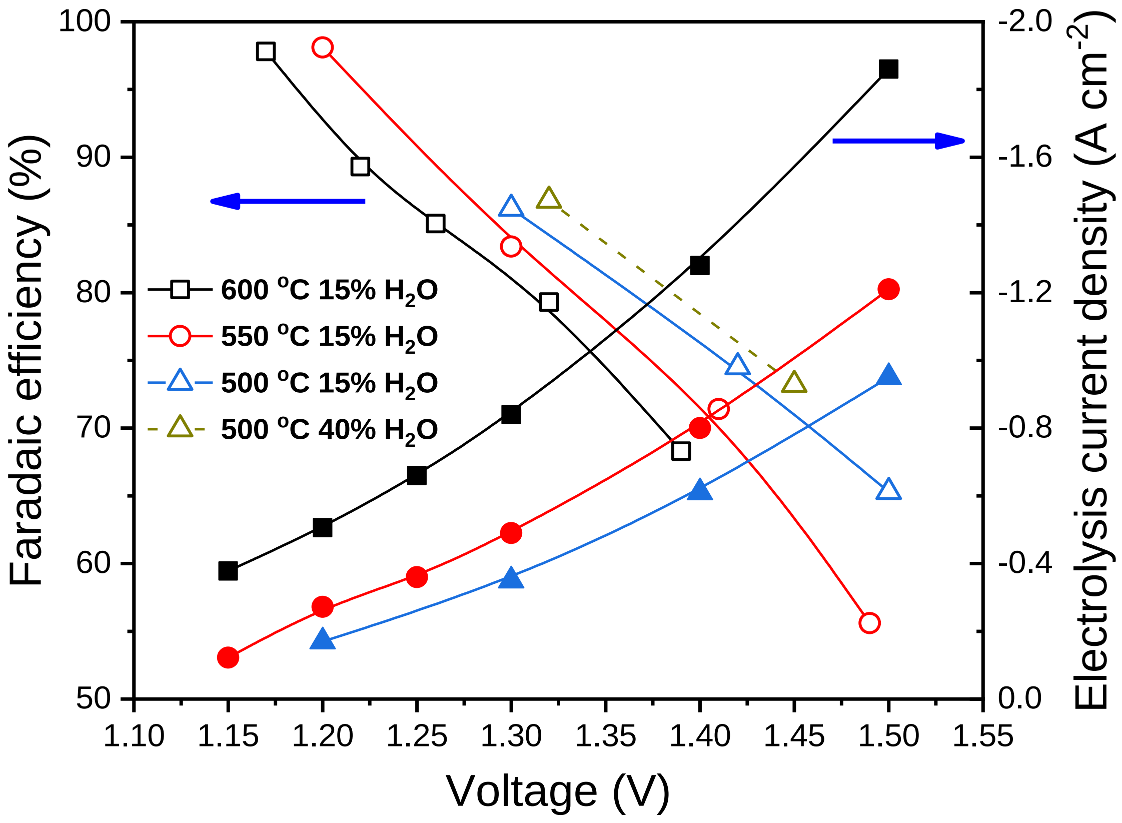

Supplementary Figure 20. Faradaic efficiency of BCZYYb4411-based PCEC. The measurements were carried out at different temperature (500~600 °C) and steam pressure (15% and 40%). In the hydrogen electrode side, dry 5% H_2 was used as feed gas. Gas chromatography was used to monitor the hydrogen concentration change at different constant current densities. The ratio of experimental and theoretical hydrogen production amounts is calculated as the efficiency.

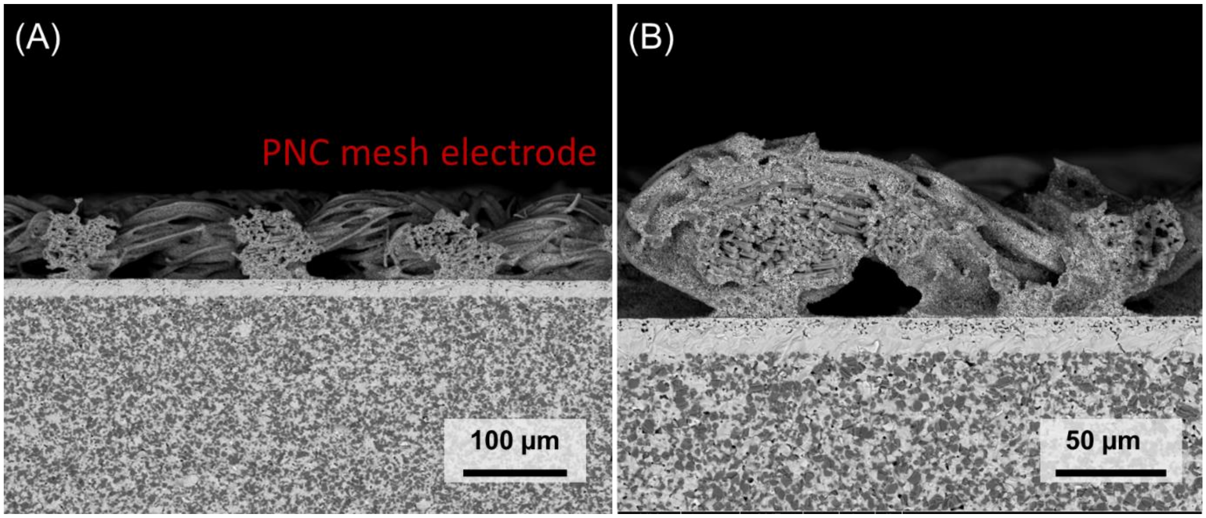

Supplementary Figure 21. Cross-sectional view of the cell with PNC mesh-like electrode. (a) single layer of nanofiber-structured mesh adhered on the electrolyte; (b) magnified view of the mechanical bonding.

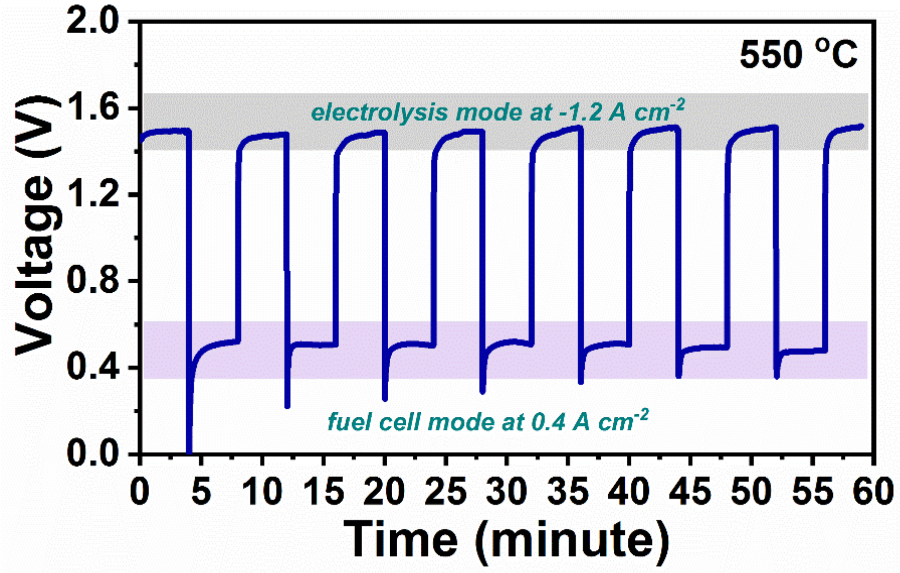

Supplementary Figure 22. Reversible operation of the electrochemical cell between electrolysis and fuel cell modes at elevated temperature of 550 °C.

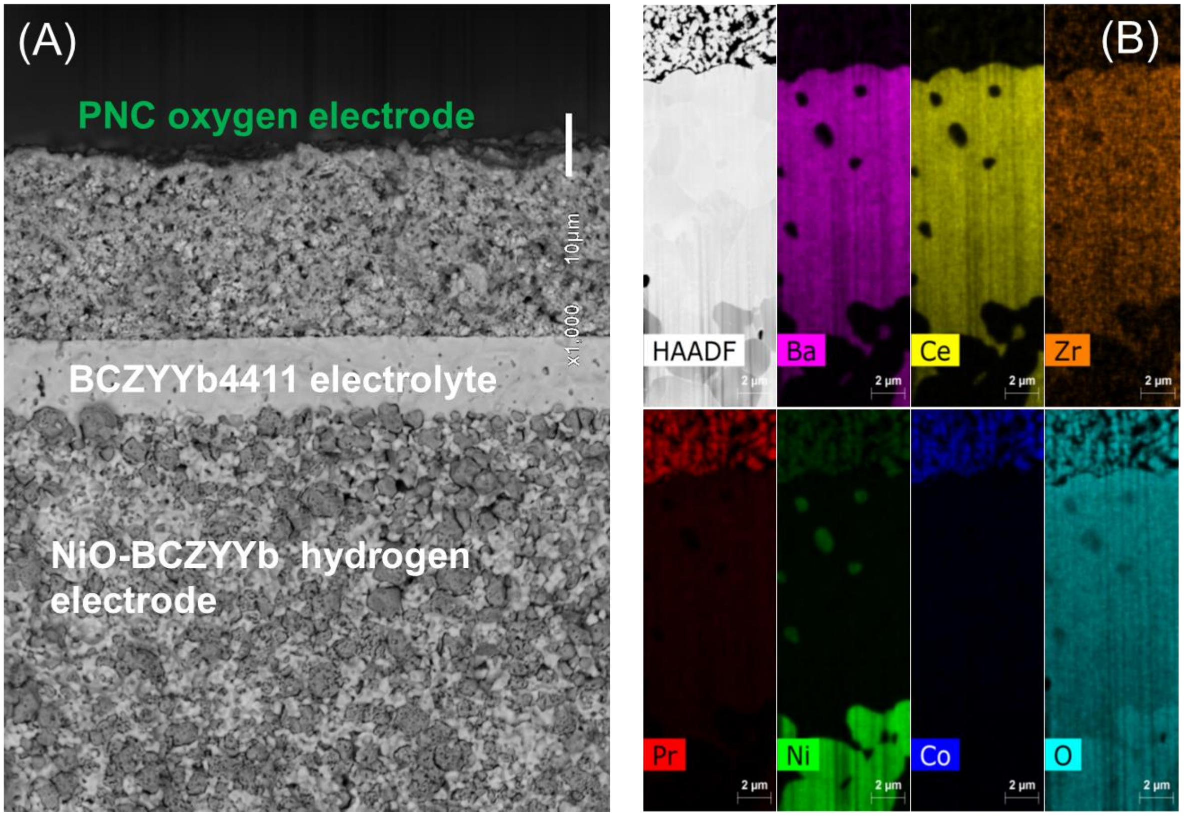

Supplementary Figure 23. Microstructure characterization of post-test PCEC with PNC oxygen electrode. (a) Cross-sectional SEM image showing three ceramic layers. (b) Focused ion beam (FIB) / TEM HAADF images and element mapping information.

Supplementary Table 1. Comparisons of electrolysis current density at different testing conditions for high-temperature and intermediate-temperature oxide-ion conducting or proton conducting electrolyzers.

Electrolyte	Steam electrode	Hydrogen electrode	T/°C	Steam ratio	Applied Voltage/V	Current /Acm^{-2}	Ref.
BZCYYb4411	3D PNC	Ni-BZCYYb4411	600<br>500<br>400	10%	1.3	1.18<br>0.56<br>0.23	This work
BZCYYb4411	Regular PNC	Ni-BZCYYb4411	600<br>500<br>400	10%	1.3	0.86<br>0.36<br>0.07	This work
BCZYYb7111	$BaCo_{0.4}Fe_{0.4}Zr_{0.1}Y_{0.1}O_{3-\delta}$	Ni-BCZYYb7111	600	10%	1.3	1.02	[54]
BCZYYb4411	PBSCF	Ni-BCZYYb4411	500	10%	1.3	0.25	[53]
$BaZr_{0.7}Ce_{0.2}Y_{0.1}O_3$	$BaGd_{0.8}La_{0.2}Co_2O_{6-\delta}$	Ni-BZCY	600	50% (1.5 bar)	1.3	0.97	[52]
BZCYYb7111	PBSCF	Ni-BZCYYb7111	600	12%	1.3	0.55	[43]
$BaZr_{0.8}Y_{0.2}O_{3-\delta}$ (BZY)	$La_{0.6}Sr_{0.4}Co_{0.2}Fe_{0.8}O_{3-\delta}$	Ni-BZY	600	3%	1.4	0.07	[42]
$BaCe_{0.5}Zr_{0.3}Y_{0.2}O_{3-\delta}$	$Sm_{0.5}Sr_{0.5}CoO_{3-\delta}$	Ni-BZCY	600	50%	1.4	0.65	[41]
$BaCe_{0.5}Zr_{0.3}Y_{0.16}Zn_{0.04}O_{3-\delta}$ (BZCYZ)	$La_{0.8}Sr_{0.2}Mn_{1-x}Sc_xO_{3-\delta}$	$La_{0.75}Sr_{0.25}Cr_{0.5}Mn_{0.5}O_{3-\delta}$ (LSCM)	700	5%	1.6	0.04	[48]
LSM-SDC-YSZ	YSZ	Ni-SDC-YSZ	850	50%	1.4	1.4	[47]
LSGM	$Ba_{0.6}La_{0.4}CoO_{3-\delta}$	$Ce_{0.6}Mn_{0.3}Fe_{0.1}O_{2-\delta}$	700	20%	1.4	0.23	[46]
$Ce_{0.9}Gd_{0.1}O_{2-\delta}$ (CGO)	$Pr_2NiO_{4+\delta}$	Ni-CGO	700	3%	1.4	0.37	[45]
YSZ	LSM-YSZ	Ni-YSZ	700	33%	1.3	0.4	[40]
$Zr_{0.88}Sc_{0.22}Ce_{0.01}O_{2.11}$ (SSZ)	$Nd_2NiO_{4+\delta}$	Ni-$Zr_{0.92}Y_{0.16}O_{2.08}$	700	50%	1.3	0.44	[39]
LSGM	PBSCF	$PrBaMn_2O_{5+\delta}$	700	10%	1.3	0.5	[44]

Supplementary Note 1: Characterization of PNC electrode.

The PNC perovskite was synthesized by a modified glycine-nitrate combustion method. The phase structure was confirmed to be a single perovskite with peaks corresponding to cobaltite $PrCoO_3$, as shown in Supplementary Figure 1a. The existence of several weak diffraction peaks suggests the PNC powder do not crystallize in a cubic or rhombohedral structure. Instead, the diffraction peaks can be readily indexed to a pure orthorhombic perovskite phase, in a good agreement with the data in ICSD (No. 89-8415) for $PrCoO_3$ *(1)*. The XRD pattern was also studied by Rietveld refinement using the GSAS program. As shown in Supplementary Figure 2, the PNC was indexed as orthorhombic, space group *Pbnm*, with a=5.405 Å, b=5.380 Å, and c=7.617 Å. To examine the chemical stability of PNC against steam, the powder was calcined at 600 °C in humid air (~50% H_2O) for 200 h. The diffraction pattern didn't show any observable changes after the test, indicating neglected interaction between PNC and steam at operating temperatures. It should be noted that the slight systematic peak shift is attributed to sample displacement in two measurements.

The morphology of the synthesized PNC powder was examined by transmission electron microscopy (TEM). The particles are only about 20~100 nm with uniform size distribution (Supplementary Figure 1b) and the Brunauer-Emmett-Teller (BET) result shows it is 147 m^2/g. The element contents in the nanoparticles as determined by an energy dispersive X-ray (EDX) analysis equipped in TEM showed the existence of Pr, Ni and Co (Supplementary Figure 1c). In Fig. S1D, the lattice-resolved high resolution TEM (HRTEM) image of the grain edge shows the presence of highly crystalline nature, which corresponds to the (101) crystal plane of perovskite structure with a lattice inter-planar spacing of d_{101}=0.387 nm. The selected area electro diffraction (SAED) pattern of boxed area confirms the long-range order crystal structure. The line element scan indicates the homogenous distribution of three cations in the particle tip (Supplementary Figure 1e-f).

The oxygen non-stoichiometry was determined by iodometric titration method combining with TGA result. As temperature increased, the oxygen deficiency was slightly reduced, indicating the slow oxidation upon the oxidizing condition (Supplementary Figure 3). As PCEC is operated between 500 °C and 600 °C in this work, the oxygen non-stoichiometry is about ~0.08. The chemical compatibility between PNC electrode and BCZYYb electrolyte was confirmed by XRD after calcining the mixture powder with 50:50wt% ratio at 1000 °C in air for

5 h. There were not any observable impurity peaks in the pattern (Supplementary Figure 4). The surface of PNC particles was examined by X-ray photoelectron spectroscopy (XPS) to find out the co-existence of Pr, Ni, Co and O elements (Supplementary Figure 5a). The valences of each metals were detected and analyzed. Specifically for Ni dopant, there are two main +2 and +3 valences found with major contributing to +2, indicating the formation of intrinsic oxygen vacancy with considerable concentration (Supplementary Figure 5b-5d).

Supplementary Note 2: DFT calculations

In the orthorhombic *Pbnm* structure of PCO before doping, Pr and Co atoms occupy the Wyckoff 4c (0.4925, -0.0371, 1/4) and 4a (0, 0, 0) positions, respectively. Oxygen atoms occupy both the Wyckoff 4c (0.5723, 0.5088, 1/4) and Wyckoff 8d (0.2827, 0.2172, 0.4608) positions, which are denoted as O1 and O2 sites, respectively. For the equilibrium lattice constants of PCO (Supplementary Figure 6a), the GGA+U calculations give *a*=5.42 Å, *b*=5.41 Å, and *c*=7.64 Å, in very good agreement with experimentally measured values (*a*=5.38 Å, *b*=5.34 Å, and *c*=7.58 Å) *(2)*. In modeling PNC structure, four Ni atoms are distributed among eight available Co sites in a 40-atom supercell such that Ni and Co atoms form a pseudo-rock salt lattice, as shown in Supplementary Figure 6b. Due to symmetry-lowering as a consequence of Ni substitution, the original Wyckoff 8d sites for oxygen in PCO split into two symmetrically distinct Wyckoff 4e sites, hereinafter referred to as O2 and O3 sites, respectively. We obtain the vacancy formation energies in different lattice oxygen positions (O1 and O2 for PCO; O1, O2, O3 for PNC) by removing a single neutral oxygen atom from the 160-atom PCO or PNC supercell. The formation energies of oxygen vacancies are calculated as $\Delta H_f^{Va} = E_{defect} - E_{perfect} + \frac{1}{2}E_{O_2}$, where E_{defect} and $E_{perfect}$ are the total energy of the 160-atom supercell after and before oxygen removal, respectively. E_{O2} is the energy of a spin polarized O_2 molecule in its ground state triplet state. Our final GGA+U results are reported in Supplementary Figure 6c.

With 50% replacement of Co sites with Ni atoms, the oxygen vacancy formation energy can be significantly decreased, e.g., from 3.40 eV to 1.61 eV at O1 sites and from 3.49 eV to 1.72 eV at O2 sites. Importantly, since the formation of oxygen vacancies is a prerequisite for water dissociative incorporation into the defective lattice, the formation energy reduction may induce essential water hydration. One should stress that LSCF oxide is an oxygen vacancy-rich material. Evidently, a large oxygen deficiency is not the only requirement to obtain an efficient proton

conductor. So far, our DFT calculations assume a non-magnetic (NM) state for both PCO and PNC. To explore the effects of magnetism, we have further performed collinear spin-polarized GGA+U calculations to find the magnetic ground states of PCO and PNC. Our calculations consider a total of eight possible magnetic configurations, including ferromagnetic (FM), antiferromagnetic (AFM), and ferrimagnetic (FI) orderings, and different initial magnetic moments to initiate the self-consistent electronic iterations. Our calculations show that PCO and PNC exhibit an AFM and FM ground states, respectively. In the case of PCO, the experimentally determined NM ground state is not determined as the exact magnetic ground state by our GGA+U calculations. The origin of this apparent discrepancy may be due to our choice of exchange-correlation functional as well as U-J value, which deserves future investigations. As shown in Supplementary Figure 7 below, PCO in both the NM and AFM configurations are semi-conductors with a band gap of 1.12 eV and 1.25 eV, respectively. Interestingly, while NM PNC is predicted to be an electron conductor due to the finite DOS at the Fermi level, FM PNC is semiconducting with a band gap of 1.05 eV.

As shown in Supplementary Figure 6d, the calculated oxygen vacancy formation energies in both PCO and PNC strongly depend on their magnetic structure. For PCO, the oxygen vacancy formation energy at O1 (O2) sites is significantly reduced from 3.40 (3.49) eV to 2.52 (2.36) eV after taking magnetism into account. In comparison, the effects of magnetism on the oxygen vacancy formation energies in PNC are much weaker, especially for the O2/O3 sites. Importantly, our spin-polarized DFT calculations also suggest that Ni doping will reduce the formation energies of oxygen vacancies, with agrees with our non-spin-polarized DFT calculations. As shown in Supplementary Figure 8, proton migration barriers in PCO and PNC also depend on the choice of magnetic structure used in DFT calculations. Consistent with our non-magnetic DFT results (Figure 2E in the main text), our spin polarized DFT calculations also suggest a reduction of proton migration energy due to Ni doping, although to a lesser degree.

Supplementary Note 3: Hydration behavior of PNC oxide observed in high-temperature X-ray diffraction and thermogravimetry analysis.

The phase structure of PNC electrode powder sample was monitored during the process of changing air humidity from dry to wet (~3% H_2O) to observe the chemical expansion due to insertion of water into the crystal structure. The proton defects ($OH_O^{\cdot}$) are formed in the oxides

by the Wagner hydration mechanism (Equation 1) in which water molecule combines with oxygen vacancy to generate two mobile protons in the anion sublattice.

$$H_2O + V_{\ddot{O}} + O_O^{\times} \leftrightarrow 2OH_{\dot{O}} \qquad (1)$$

The chemical expansion due to hydration process can be observed by examining the lattice parameter with XRD (Supplementary Figure 9). At 600 °C, when the gas was switched from dry air to wet air, the clear shift of diffraction peaks towards left side can be observed, indicating the expansion of crystal structure.

The hydration behavior of the electrode in wet air condition was also observed in TGA examination, as shown in Supplementary Figure 10. The powder was firstly heated up to 950 °C with ramping rate of 5 °C/min in dry air to remove the surface residues. After cooling to 500 °C and reaching the equilibrium, the wet air was flushed into the chamber to start the hydration process. The weight increase was recorded as function of hydration time as total time is 15 h. The weight gain after hydration for PNC is found to be highest ~0.055%, comparing to 0.048% for PBSCF, and 0.031% for LSCF, respectively.

Results from hydrogen permeation experiment are meaningful to demonstrate the presence of mixed proton and electron conduction. As well known, dense membranes for hydrogen permeation based on high temperature proton conductors have been studied for many years due to mixed electron and proton conductivity in ceramic-metal or dual ceramic-ceramic composites. About the concern of hydrogen permeation through grain boundary, it is common to see that grain-boundary diffusion could contribute an appreciable amount to the overall permeation rate in palladium and its alloys, particularly in the case of a nanostructured membrane *(3)*. In ceramic samples, recently there are several studies carried out to investigate the importance and dependence of gas permeation on the microstructure of mixed conducting oxides. Whether the transport along grain boundaries displays a barrier or acts as a pathway for fast transport is not clearly determined. For example, Diethelm et al. found for $La_{0.5}Sr_{0.5}FeO_{3-\delta}$ that the larger the obtained grains, and thus the fewer the grain boundaries, the smaller the observed permeation performance *(4)*. Similar findings were also reported for $SrCo_{0.8}Fe_{0.2}O_{3-\delta}$ membranes by Zhang et al. who observed that increasing grain size by increasing sintering temperature resulted in a considerable decrease in permeation flux *(5)*. Contrary to these findings, for the ceramics $LaCoO_{3-}$

$_{\delta}$, $La_{0.3}Sr_{0.7}CoO_{3-\delta}$, $La_{0.6}Sr_{0.4}Co_{0.2}Fe_{0.8}O_{3-\delta}$, $Ba_{0.5}Sr_{0.5}Co_{0.8}Fe_{0.2}O_{3-\delta}$ and $CaTi_{0.8}Fe_{0.2}O_{3-\delta}$, the increase of grain size leads to an enhanced permeation *(6-10)*. In this work, we agree that hydrogen permeation through grain boundary is possible, as we can see very small amount of hydrogen flux can be measured in dry 3% H_2, e.g., 2.6×10^{-8} mol/cm^2 s at 400 °C. However, the hydrogen flux was significantly increased by 5 times when PNC is exposed to wet gas, which indicates the hydration process introduced effective proton conductivity that facilitated permeation flux.

Supplementary Note 4: Fourier-transform infrared spectroscopy (FTIR) and temperature programmed desorption (TPD) techniques for detecting proton defects in PNC.

To further demonstrate the presence of protons in the PNC lattice, two other techniques were employed to measure the water signal during the dehydration process. For FTIR examination, the PNC powder sample was firstly hydrated at 600 °C in wet condition and then cooled down to room temperature. After flushing with dry Ar for 1 hour, the background spectrum was collected. The FTIR spectra were then collected every 50 °C from 50 °C to 600 °C in dry Ar with a ramping rate of 10 °C/min. The negative OH peak intensity (3000-3500 cm^{-1}) of PNC increased monotonously with temperature (Supplementary Figure 11) *(11)*, indicating the continuous loss of hydroxyl groups on the PNC surface. The negative peak intensity increased rapidly before 400 °C and then smoothly after 400 °C, which indicates two different temperature zones for the desorption of surface chemisorbed hydroxyl/water (<400 °C) and lattice protons (>400 °C) respectively.

Temperature programmed desorption (TPD) is rarely used to investigate desorbed water molecules from surface and dehydration process in the bulk material when the temperature is increased *(11)*. It is considered that the desorption of water on the particle surface can occur at temperature around 100~300 °C and the water released at higher temperature range (> approximate 400 °C) is from the dehydration process. Therefore, this PNC material is examined by TPD method. The powder was firstly hydrated at 600 °C for 2 h in ~10% H_2O/air) and then cooled down to room temperature. The PNC powder was then reheated in dry air and mass spectroscopy was used to monitor the real-time water signal. As shown in Supplementary Figure 12, two samples were examined to reproduce the result. As expected, the residual water started to desorb at low temperature (<300 °C) and when the temperature reached the range of 500 to 700 °C a weak but discernable broad peak appeared which could be attributed to the water formation from the dehydrated proton defects in the bulk.

Supplementary Note 5: Chemical stability in hydrogen permeation experiment and reducing condition.

The chemical stability of PNC oxide after hydrogen permeation experiment was examined (Supplementary Figure 13a). The XRD pattern indicated no any impurities after pellet sample exposing to 3% H_2 at 500 °C. In addition, the chemical stability under more reducing condition is evaluated at different hydrogen concentration (3%, 20%, 50%, and 100%) and temperature (500~700 °C) (Supplementary Figure 13b). As can be seen, PNC is stable against diluted 3% H_2 when the temperature is increased up to 700 °C; however, when hydrogen centration is increased to 20%, PNC can decompose into oxides at 700 °C. The decomposition also occurs in the higher H_2 concentration. Therefore, there is some limitation on atmosphere when this material is applied for hydrogen permeation membrane.

Supplementary Note 6: Electrochemical impedance spectra at reduced temperatures (400~600 °C).

The impedance spectra in fuel cell mode under open circuit conditions are shown in Supplementary Figure 14a. When the operating temperature was decreased from 600 °C to 500 °C, both ohmic and electrode polarization resistances increase correspondingly. The activation energy for electrolyte resistance was obviously smaller than that of interfacial electrode polarization. In electrolysis mode, when the operating temperature of the cell was decreased to lower-temperature range, the electrode polarization resistance was 0.35 Ω cm^2 at 450 °C and 0.6 Ω cm^2 at 400 °C, respectively (Supplementary Figure 14b). The total cell resistance at 400 °C was even smaller than recent reported BCZYYb-based electrolysis cell (1.32 Ω cm^2) at higher temperature of 500 °C with 3D self-architectured PBSCF as steam electrode.

Supplementary Note 7: Hydrogen production in different conditions.

Supplementary Figure 15 shows the dependence of electrolysis performance on oxygen partial pressure at oxygen electrode side and hydrogen concentration at hydrogen electrode side at 500 °C. When the humid air was switched to oxygen, the cell showed higher current density at the same electrolysis voltage. The results are consistent with the finding in the study of symmetric cell, whereas higher oxygen partial pressure can improve electrode polarization resistance (Supplementary Figure 16). In contrast, it exhibited much poor performance in argon which is opposite to the expectation that low partial pressure can promote water splitting reaction

by removal of product. At hydrogen electrode side, the less concentrated hydrogen gas enhanced the hydrogen production, and in pure argon the cell showed the highest current density.

Supplementary Note 8: Long-term stability of material structure and electrode activity.

Supplementary Figure 17 shows the impedance spectra for the cell before and after the long-term stability testing shown in Figure 3E. The high frequency ohmic resistance and electrode polarization resistance are both decreased after the testing, e.g., from 0.46 Ω cm^2 to 0.445 Ω cm^2. The result demonstrates the material and interface stability over electrolysis reaction. The direct evidences were obtained from the chemical stability of PNC electrode and BCZYYb electrolyte and activity stability of symmetric cell (Supplementary Figure 18). The XRD patterns reveal no chemical reaction between PNC/BCZYYb and steam at operating temperature for 500 h. Furthermore, the electrode polarization resistances at elevated water pressure (10%, 20% and 50% H_2O) at 500 and 600 °C were measured over respective few hundred hours. Under 20% water pressure, PNC electrode shows stable resistance indicating strong electrode/electrolyte interfacial bonding. At 50% H_2O, the electrode resistance still needs improvement as some degradation can be observed at the beginning of the testing. To examine the long-term stability of the cell with PNC electrode, another testing was run at 1.4 V, 20% H_2O and 500 °C to observe the degradation over time (Supplementary Figure 19). At the beginning, the electrolysis current density decreased slightly but then stabilized over the next 480 hours. The operation at lower temperature of 500 °C is more favorable for stable electrolysis because the materials and interfaces are more stable.

Supplementary Note 9: Faradaic efficiency in BCZYYb4411-based PCEC at different operating conditions.

The Faradaic efficiencies at different temperatures (500~600 °C) and steam concentrations were measured by on-line GC to monitor the change of hydrogen concentration in the gas flow of hydrogen electrode. It is evident that the efficiency was affected by the operating temperature and steam concentration. As operating temperature was decreased, at the same concentration of 15% the Faradaic efficiency was improved slightly at the fixed electrolysis voltage. It is attributed to the increased proton transfer number at lower temperature while the hole conductivity tends to be eliminated and the reduction of cerium ions are also diminished. In addition, when the steam concentration was increased, the efficiency can be increased. The

higher steam concentration can increase the hydration of electrolyte and decrease the material oxidation of creating more holes.

Supplementary Note 10: Incorporation of 3D mesh-like PNC electrode into PCEC.

The 3D PNC mesh electrode was optimized by firing it at different temperature: 600, 700, 800 and 900 °C respectively to obtain the good balance among porosity, performance and mechanical strength. Firstly, the PNC precursor solution was prepared by dissolving stochiometric amounts of nitrates in distilled water. A fabric textile (Telio, Montreal, CA) was immersed in the precursor solution for 24 h, followed by firing at different temperature for 4 h with a heating rate of 1 °C min^{-1} to form PNC ceramic textile. The result showed the highest performance for the electrode firing at 800 °C with favorable strength. Supplementary Figure 21 shows the cross-sectional view of the cell with nanofiber structured electrode. As can be seen, the single layer of PNC mesh was adhered strongly to electrolyte thin film by PNC ink. The use of PNC ink can improve the adhesion to enhance the interfacial polarization while the high porosity is not affected. The open space between the mesh bundles can allow the direct gas diffusion without any obstruction which significantly benefits the steam/oxygen transport for reactions at entire electrode surface.

Supplementary Note 11: Performance comparisons.

To better show the comparison of water electrolysis performances, the performances of different material systems (electrolyte and steam electrode) were summarized in Supplementary Table 1. At 600 °C, the early-stage works by Traversa et al. and Peng et al. showed electrolysis activity in the low current-density range, e. g., 0.21 A cm^{-2} at 1.4 V. Kim et al. recently reported some electrolyzers based on oxide-ion conducting electrolyte or hybrid electrolyte showing promising performances. For example, a highly efficient SOEC using layered perovskites, $PrBaMn_2O_{5+\delta}$ and $PrBa_{0.5}Sr_{0.5}Co_{1.5}Fe_{0.5}O_{5+\delta}$, as both electrodes, was fabricated and evaluated to show 0.68 A cm^{-2} at 1.4 V and 0.78 A cm^{-2} at 1.5 V respectively. With a new concept of hybrid-SOEC with an electrolyte that conducts oxygen ion and proton simultaneously, recently a layered perovskite $NdBa_{0.5}Sr_{0.5}Co_{1.5}Fe_{0.5}O_{5+\delta}$-$BaCe_{0.7}Zr_{0.1}Y_{0.1}Yb_{0.1}O_3$ composite electrode was used as steam electrode. This cell at 600 °C showed 0.85 A cm^{-2}, 1.31 A cm^{-2}, and 1.9 A cm^{-2} at 1.3 V, 1.4 V, and 1.5 V, respectively. Further optimization can further improve electrolysis current

density to 1.18 A cm^{-2} at 1.3 V, 1.72 A cm^{-2} at 1.4 V and 2.35 A cm^{-2} at 1.5 V. Therefore, for PNC electrode enabled PCEC in this work, the electrolysis ability has surpassed these high-performance O-SOEC, H-SOEC, or hybrid-SOEC, which have been recently developed.

Supplementary Note 12: Self-sustainable reversible operation between electrolysis mode and fuel cell mode.

Supplementary Figure 22 shows the voltage response of the reversible cell when it was operated between electrolysis (-1.2 A cm^{-2}) and fuel cell mode (0.4 A cm^{-2}) transiently at 550 °C. The performance in electrolysis mode was stable after each fuel cell cycle, indicating nearly constant hydrogen production to supply fuel for electricity generation. In fuel cell mode, the cell also showed decreased discharging voltage similar with the case at 500 °C due to fuel deficit caused by limitation of experiment apparatus for the cell to fully react with generated hydrogen.

Supplementary Note 13: Microscopy characterization of the PCEC after test.

Supplementary Figure 23 shows the microstructure view of the cell after a series of electrochemical testing by SEM and FIB/TEM. Firstly, the cross-sectional image shows the electrode-supported cell with respective three layers. The thicknesses of PNC electrode and BCZYYb4411 electrolyte are about 50 μm and 15 μm, respectively. Both the cathode and anode frame bond well to the electrolyte without any cracks at the interfaces after the test in high water vapor, indicating good mechanical bonding and material stability in this condition, which is consistent with the stable electrolysis current densities under different applied voltages. The PNC electrode particles did not show obvious growth or agglomeration after the long-term operation. The FIB technique was used to cut a very thin piece of the three-layer sample for element mapping, and the result clearly shows the uniform distribution and no signal of element diffusion, which indicates there is no chemical reaction or migration during the long-time testing process and the electrode/electrolyte interface is robust against high steam conditions and oxygen evolution reaction at this electrode side.

Supplementary References

1 Wang, H., Li, G. S., Guan, X. F., Zhao, M. L. & Li, L. P. Lightly doping Ca^{2+} in perovskite $PrCoO_3$ for tailored spin states and electrical properties. *Phys. Chem. Chem. Phys.* **13**, 17775-17784 (2011).

2 Alonso, J. A., Martínez-Lope, M. J., Calle, C. & Pomjakushin, V. Preparation and structural study from neutron diffraction data of $RCoO_3$ (R = Pr, Tb, Dy, Ho, Er, Tm, Yb, Lu) perovskites. *J. Mater. Chem.* **16**, 1555-1560 (2004).

3 McCool, B. A. & Lin, Y. S. Nanostructured thin palladium-silver membranes: effects of grain size on gas permeation properties. *J. Mater. Sci.* **36**, 3221-3227 (2001).

4 Diethelm, S., Van Herle, J., Sfeir, J. & Buffat, P. Correlation between oxygen transport properties and microstructure in $La_{0.5}Sr_{0.5}FeO_{3-\delta}$. *J. Eur. Ceram. Soc.* **25**, 2191-2196 (2005).

5 Zhang, K., Yang, Y. L., Ponnusamy, D., Jacobson, A. & Salama, K. Effect of microstructure on oxygen permeation in $SrCo_{0.8}Fe_{0.2}O_{3-\delta}$. *J. Mater. Sci.* **34**, 1367-1372 (1999).

6 Kharton, V. V., Naumovich, E. N., Kovalevsky, A. V., Viskup, A. P., Figueiredo, F. M., Bashmakov, I. A. & Marques, F. M. B. Mixed electronic and ionic conductivity of $LaCo(M)O_3$ (M= Ga, Cr, Fe or Ni), IV. Effect of preparation method on oxygen transport in $LaCoO_{3-\delta}$. *Solid State Ionics* **138**, 135-148 (2000).

7 Arnold, M., Martynczuk, J., Efimov, K., Wang, H. & Feldhoff, A. Grain boundaries as barrier for oxygen transport in perovskite-type membranes. *J. Membr. Sci.* **316**, 137-144 (2008).

8 Wang, H., Tablet, C., Feldhoff, A. & Caro, J. Investigation of phase structure sintering, and permeability of perovskite-type $Ba_{0.5}Sr_{0.5}Co_{0.8}Fe_{0.2}O_{3-\delta}$ membranes. *J. Membr. Sci.* **262**, 20-26 (2005).

9 Zeng, P. Y., Ran, R., Chen, Z. H., Gu, H. X., Shao, Z. P., Costa da, J. C. D. & Liu, S. M. Significant effects of sintering temperature on the performance of $La_{0.6}Sr_{0.4}Co_{0.2}Fe_{0.8}O_{3-\delta}$ oxygen selective membranes. *J. Membr. Sci.* **302**, 171-179 (2007).

10 Shaula, A. L., Fuentes, R. O., Figueiredo, F. M., Kharton, V. V., Marques, F. M. B. & Frade, J. R. Grain size effects on oxygen permeation in sub micrometric $CaTi_{0.8}Fe_{0.2}O_{3-\delta}$ ceramics obtained by mechanical activation. *J. Eur. Ceram. Soc.* **25**, 2613-2616 (2005).

11 Nagao, M., Takeuchi, A., Heo, P., Hibino, T., Sano, M. & Tomita, A. A proton-conducting In^{3+}-doped SnP_2O_7 electrolyte for intermediate-temperature fuel cells. *Electrochem. and Solid-State Lett.* **9**, A105-A109 (2006).
